# A Cell-Permeable Ester Derivative of the JmjC Histone Demethylase Inhibitor IOX1

**DOI:** 10.1002/cmdc.201300428

**Published:** 2014-02-06

**Authors:** Rachel Schiller, Giuseppe Scozzafava, Anthony Tumber, James R Wickens, Jacob T Bush, Ganesha Rai, Clarisse Lejeune, Hwanho Choi, Tzu-Lan Yeh, Mun Chiang Chan, Bryan T Mott, James S O McCullagh, David J Maloney, Christopher J Schofield, Akane Kawamura

**Affiliations:** [a] Chemistry Research Laboratory, Department of Chemistry, University of Oxford Mansfield Road, Oxford, OX1 3TA (UK) E-mail: christopher.schofield@chem.ox.ac.uk akane.kawamura@chem.ox.ac.uk; [b] Structural Genomics Consortium, Nuffield Department of Medicine, University of Oxford Old Road Campus, Headington, OX3 7DQ (UK); [c] National Center for Advancing Translational Sciences (NCATS), National Institutes of Health (NIH) 9800 Medical Centre Drive, Bethesda, MD MSC 3370 (USA); [d] Wellcome Trust Centre for Human Genetics, University of Oxford Roosevelt Drive, Oxford, OX3 7BN (UK)

**Keywords:** 2-oxoglutarate (2OG) oxygenases, cell permeability, epigenetics, inhibitors, jmjc histone demethylases, structure–activity relationships

## Abstract

The 2-oxoglutarate (2OG)-dependent Jumonji C domain (JmjC) family is the largest family of histone lysine demethylases. There is interest in developing small-molecule probes that modulate JmjC activity to investigate their biological roles. 5-Carboxy-8-hydroxyquinoline (IOX1) is the most potent broad-spectrum inhibitor of 2OG oxygenases, including the JmjC demethylases, reported to date; however, it suffers from low cell permeability. Here, we describe structure–activity relationship studies leading to the discovery of an *n*-octyl ester form of IOX1 with improved cellular potency (EC_50_ value of 100 to 4 μm). These findings are supported by in vitro inhibition and selectivity studies, docking studies, activity versus toxicity analysis in cell cultures, and intracellular uptake measurements. The *n*-octyl ester was found to have improved cell permeability; it was found to inhibit some JmjC demethylases in its intact ester form and to be more selective than IOX1. The *n*-octyl ester of IOX1 should find utility as a starting point for the development of JmjC inhibitors and as a use as a cell-permeable tool compound for studies investigating the roles of 2OG oxygenases in epigenetic regulation.

Epigenetic processes regulate gene expression in a context-dependent manner by reversible modifications to chromatin.[1] An extensive literature documents a wide range of post-translational histone modifications or “marks” that regulate chromatin accessibility, including acetylation and methylation.[2] Histone lysine methylation can activate or repress transcription, depending on the site and the extent of modification. Some methylation marks, such as trimethylation of histone-3 lysine-4 (H3K4me3), are associated with transcriptional activation, whereas other marks, such as H3K9me3, are primarily associated with transcriptional repression.[3] Although histone methylation was once considered irreversible, it is now known that, like acetylation, it is reversible, opening the opportunity for pharmaceutical intervention.[4]

Two classes of histone lysine demethylases (KDMs) have been identified, which differ in their catalytic mechanisms. The lysine-specific demethylases (LSD) employ a flavin-mediated demethylation.[5] In contrast, the larger class of Jumonji C domain (JmjC) demethylases catalyse demethylation via initial methyl group hydroxylation (Scheme 1). The JmjC demethylases belong to the superfamily of Fe^II^ and 2-oxoglutarate (2OG) oxygenases.[6] In contrast to the LSD KDMs, JmjC KDMs accept all three methylated forms of lysine; their reported substrate residues include H3K4, H3K9, H3K27 and H3K36.[7] More than 30 human JmjC oxygenases have been identified, some of which are demethylases with the remainder being hydroxylases.[8, 9] Most of the JmjC proteins contain auxiliary functional domains, such as prolyl hydroxylase (PHD), Tudor and Zn^II^ finger domains, which are likely to contribute to substrate selectivity.[10, 11] Dysregulation of JmjC demethylases can lead to aberrant histone methylation states and is associated with a number of diseases, including cancer and neurological disorders such as autism and X-linked mental retardation (XLMR).[12–17] These findings advocate further investigations into the mechanisms by which these KDMs work, and the development of small-molecule chemical probes as tools to evaluate their therapeutic potential.

**Scheme 1:**
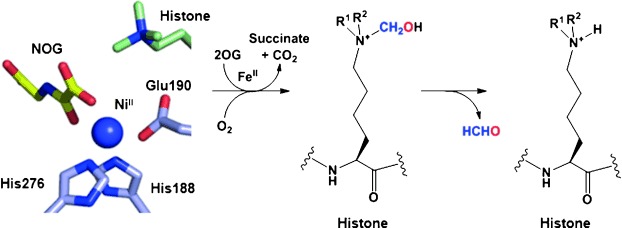
Schematic mechanism for the demethylation of methyl-lysine histone by JmjC catalysis. JmjC active site residues for Fe^II^ coordination are taken from a crystal structure of human KDM4A in complex with histone H3 peptide trimethylated at Lys 9 (PDB: 2OQ6[22]); Ni^II^ and *N*-oxalylglycine (NOG) are substitutes for Fe^II^ and 2OG, respectively.

A chemical probe approach offers an advantage over genetic techniques in validating epigenetic targets as it enables targeting of individual domains.[18] Moreover, small-molecule inhibitors can be administered in a reversible, dose-dependent manner, whereas the use of genetic methods is currently less controllable. Advances in understanding the enzymatic mechanisms and structural elucidation of the JmjC demethylases have permitted the identification of small-molecule inhibitors, and examples of commonly used 2OG oxygenase inhibitors are shown in Figure 1.[19–21]

**Figure 1:**
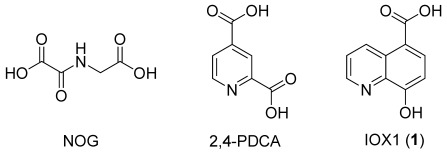
2-Oxoglutarate (2OG) analogues reported as broad-spectrum histone lysine demethylase (KDM) inhibitors: *N*-oxalylglycine (NOG), and 2,4-pyridinedicarboxylic acid (2,4-PDCA), IOX1 (1).

Of these broad-spectrum inhibitors, IOX1 (**1**) is reported to be the most potent against a representative panel of 2OG oxygenases, including non-JmjC 2OG oxygenases, with an in vitro IC_50_ value in the micromolar range. However, its efficacy in cells is about a hundred-fold lower (HeLa cells, KDM4A, IC_50_=86 μm), possibly due to low cell permeability resulting from its polar C-5 carboxyl group.[23]

With the aim of improving the transmembrane permeability of IOX1, ester derivatives with different lengths of alkoxy groups were synthesised (Table 1). Methods for the synthesis of 5-carboxy-8-quinolinol derivatives have been reported for various uses.[24–26] The Skraup reaction was employed to synthesise the quinoline IOX1 (**1**) from 3-amino-4-hydroxybenzoic acid and acrolein. The ethyl (**3**), *n*-butyl (**4**) and *n*-octyl (**5**) ester derivatives were prepared by Fischer esterification. Methyl ester **2** was synthesised using 5-bromoquinolin-8-ol employing organopalladium chemistry. To test whether improved permeability could be obtained by substitution of both phenol and carboxylic acid groups of IOX1, methyl acetate diester **6** was produced from **2** and acetic anhydride in the presence of catalytic 4-dimethylaminopyridine. Branched diester derivative **7** was synthesised using the conditions reported by Nudelman and co-workers.[27, 28]

**Table 1:** Structure–activity relationships for IOX1 (1) and its ester derivatives 2–7

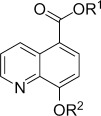					
Compd	R^1^	R^2^	CC_50_^[a]^ [μm]	EC_50_^[b]^ [μm]	IC_50_^[c]^ [μm]
**1**	H	H	>300	100.0	0.6
**2**	CH_3_	H	10	50.0	10.7
**3**	CH_2_CH_3_	H	66	>100	14.9
**4**	(CH_2_)_3_CH_3_	H	50	22.0	5.0
**5**	(CH_2_)_7_CH_3_	H	>300	3.8	3.9
**6**	CH_3_	COCH_3_	29	>100	10.5
**7**	CH_2_OCOC(CH_3_)_3_	CH_2_OCOC(CH_3_)_3_	17	–	>100

[a] CC_50_ values derived from HeLa cell viability assays. [b] EC_50_ values derived from immunofluorescence assays of KDM4A activity in HeLa cells. [c] IC_50_ values derived from AlphaScreen assays of isolated KDM4C. Data represent the mean of *n*≥3 replicates (Figures S1, 2, and 5 in the Supporting Information).

The viability of HeLa cells was analysed after 24 h following incubation with different concentrations (1–300 μm) of IOX1 (**1**) or its ester derivatives **2**–**7** (Table 1; Figure S1 in the Supporting Information). Methyl ester derivative **2** was the most cytotoxic compound, with a CC_50_ value of 10 μm. Di-substituted compounds **6** and **7** had CC_50_ values of 29 and 17 μm, respectively. Ethyl **3** and *n*-butyl **4** esters had similar CC_50_ values of 50 and 66 μm, respectively. Out of the tested compounds, only **7** resulted in complete toxicity at the highest concentration tested, while treatment with the other compounds led to between 25 % and 60 % viable cells. *n-*Octyl ester **5** was not cytotoxic in the tested concentration range, with a CC_50_ value greater than 300 μm and with over 50 % viable cells at the highest concentration tested. This CC_50_ value is similar to the CC_50_ value obtained here for IOX1 (**1**) (>300 μm), which is in agreement with the reported value for IOX1 (292 μm).[21]

Immunofluorescence assays were then used to assess the effect of IOX1 ester derivatives on demethylation activity in cells using KDM4A as a representative JmjC KDM.[21] Flag-tagged KDM4A was transiently overexpressed in HeLa cells, and these were then treated with either a vehicle control (DMSO) or varying concentrations (1–300 μm) of IOX1 (**1**) or IOX1 ester derivatives **2**–**7**. After 24 h of compound dosing, the cells were analysed by indirect immunofluorescence using an anti-Flag tag antibody to identify cells overexpressing KDM4A, and an antibody for endogenous H3K9me3 to quantify the level of this histone modification, known to be regulated by KDM4A.[7] As a control, cells overexpressing the H188A catalytically deficient KDM4A variant were also analysed. Treatment with increasing concentrations of IOX1 (**1**) or the ester derivatives caused a dose-response-dependent increase in H3K9me3 fluorescence intensity, implying KDM4A inhibition in cells by direct or indirect mechanisms (Figure 2). The cellular EC_50_ value of **1** was determined to be 100 μm (Table 1), correlating with the reported value (EC_50_=86 μm).[23] The apparent cellular EC_50_ values of derivatives **2**, **4** and **5** were substantially lower than that of IOX1 (**1**), indicating better inhibition of KDM4A activity (Figure S2 in the Supporting Information). The most potent derivative was *n-*octyl ester **5** was approximately 30-fold more active than IOX1 (**1**), with an EC_50_ value of 3.8 μm (Figure 2).

**Figure 2:**
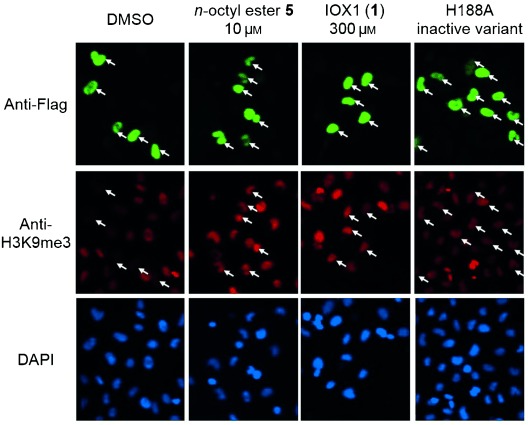
*n*-Octyl ester 5 increases H3K9me3 levels in HeLa cells via KDM4A inhibition. Indirect immunofluorescence assays with anti-Flag (green) and anti-H3K9me3 (red) antibodies and with DAPI staining (blue) in HeLa cells overexpressing Flag-tagged KDM4A. DMSO treatment has no effect on KDM4A demethylase activity, while IOX1 (1) (300 μm) and *n*-octyl ester 5 (10 μm) treatment resulted in increased H3K9Me3 substrate levels (white arrows indicate transfected cells). The H188A catalytically inactive KDM4A variant does not affect H3K9Me3 levels.

An intracellular delivery assay was then performed to compare the cell permeabilities of compounds **1** and **5**, as well as to investigate the level of hydrolysis of **5** to the parent compound (**1**) in cells. HeLa cells were dosed with IOX1 (**1**) or *n*-octyl ester **5** at a concentration of 200 μm and incubated for 24 h. The intracellular levels of the two forms were then analysed by LC-MS/MS. Relative quantitative data were collected, and the compounds were identified by comparing mass and retention times with values for standards. The *n*-octyl ester (**5**) was found to be more abundant in cells than IOX1 (**1**) (∼sixfold) indicating better cell permeability for **5** than **1** (Table 2; Figure S3 in the Supporting Information). Only a small fraction (∼2 %) of **5** was observed to be hydrolysed to the parent compound IOX1 (**1**). Together with the activities detected in the cellular assay and with isolated proteins (Table 3), the results of the intracellular delivery assay indicate that the unhydrolysed form of **5** accounts for at least some of the KDM inhibitory activity observed in cells treated with ester **5**.

**Table 2:** Intracellular delivery of IOX1 (1) and *n*-octyl ester 5^[a]^

Dosed compd	Lysate concentration [fmol cell^−1^]	
	IOX1 (1)	*n*-Octyl ester (5)
IOX1 (**1**)	0.624±0.134	0.030±0.001
*n*-Octyl ester **5**	0.080±0.006	4.083±1.290

[a] IOX1 (**1**) and *n*-octyl ester **5** were detected in the lysates of HeLa cells 24 h after the administration of IOX1 (**1**) or *n*-octyl ester **5** at a concentration of 200 μm; data represent the mean±SD of *n*=3 replicates.

Interestingly, in cell experiments with **5** analysing for upregulation of the alpha-subunit of the hypoxia-inducible transcription factor (HIF) by inhibition of the 2OG-dependent HIF hydroxylases, an increase in HIF levels was observed in cells treated with **5** (Figure S4 in the Supporting Information).[8] While ester **5** is a relatively poor PHD inhibitor (Table 3), it is possible that hydrolysis of **5** results in a sufficient amount of **1** to cause PHD inhibition in cells. However, it is also possible that the HIF upregulation is in part mediated by inhibition of 2OG oxygenases other than PHDs, or by other mechanisms. Overall, it seems likely that both the hydrolysed (i.e., IOX1) and nonhydrolysed forms of **5** contribute to cellular activities.

On the basis of crystallographic analysis, the C-5 carboxylic acid of IOX1 was proposed to be important for active site binding, therefore it might be expected that the ester derivatives would be substantially less potent than IOX1.[29] To test this proposal, we assayed the ability of the compounds to inhibit the H3K9me3 demethylation activity of isolated KDM4C using an amplified luminescent proximity homogeneous assay (ALPHA) screen.[30] For IOX1 (**1**), an IC_50_ value of 0.6 μm was obtained, identical to that reported in the literature (Table 1; Figure S5 in the Supporting Information).[23] Apart from the bulky di-*tert*-butyl diacetate derivative, **7**, the esters displayed similar activities in the micromolar range, with **5** being the most potent (IC_50_=3.9 μm). *n-*Octyl ester **5** was shown to be stable to hydrolysis in the AlphaScreen buffer according to LC-MS analysis (Figure S6 in the Supporting Information). The activity of derivative **5** and of the other esters, as determined by the AlphaScreen assay, indicates that the C-5 ester derivatisation can be tolerated, while preserving some KDM inhibitory activity.

IOX1 analogues with lipophilic substitution of the C-5 carboxylic acid have been reported to inhibit JmjC proteins.[21, 31] Docking simulations were performed to explore the rationale behind the structure–activity relationships observed in the AlphaScreen assays (Table 1). These simulations included IOX1 esters, with linear alkyl chains ranging in length between one and ten carbons, docked into the X-ray crystal structure of the KDM4A active site in complex with IOX1 (PDB: 3NJY[21]). The docking results indicate that the KDM4A active site can accommodate IOX1 ester derivatives including *n*-octyl and even *n*-decyl esters (Figure 3; Figure S7 in the Supporting Information). In agreement with the AlphaScreen results, IOX1 exhibited the strongest predicted binding to the active site as deduced by the calculated Gibbs free energy (Δ*G*=−7.05 Kcal mol^−1^; Table S1 in the Supporting Information). The shorter esters, with one or two carbons, had IC_50_ values of >10 μm in the AlphaScreen and calculated Δ*G* values of greater than −6.5 Kcal mol^−1^ indicating weaker binding compared with IOX1. The longer esters, with three to ten carbons, had IC_50_ values of ≤5 μm in the AlphaScreen and calculated Δ*G* values lower than −6.5 Kcal mol^−1^. This improved binding indicated by the docking simulations correlates with higher potency in the AlphaScreen and could be explained by a hydrophobic effect. Increasing the length of the alkyl chain is likely to increase the binding affinity to the hydrophobic region leading to the active site, where the aliphatic ester chain is accommodated. These docking observations combined with the structure–activity data may be useful in the structure-based identification of new JmjC inhibitors.

**Figure 3:**
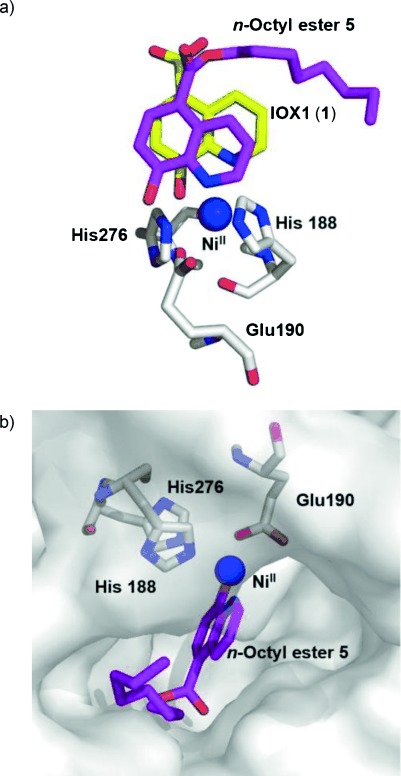
Docking of *n*-octyl ester 5 in the KDM4A active site using a crystal structure of KDM4A bound to IOX1 (PDB: 3NJY[21]). a) Overlay of the docked position of *n*-octyl ester 5 (pink) with that observed for IOX1 (yellow); b) Surface view of modelled 5 in the active site pocket.

A more extended set of AlphaScreen assays were then used to compare the selectivity of *n*-octyl ester **5** with that of IOX1 (**1**) and the shorter ester derivatives (methyl ester **2** and *n*-butyl ester **4**) against additional 2OG oxygenases. The assays were performed using representatives of different JmjC KDM subfamilies (KDM4C, KDM4E, KDM2A, KDM3A, KDM5C and KDM6B) and the catalytic domain of a HIF prolyl hydroxylase (PHD2). The results support the classification of IOX1 (**1**) as a broad-spectrum 2OG oxygenase inhibitor, with IC_50_ values in the micromolar range against all of the tested oxygenases (Table 3; Figure S8 in the Supporting Information).[23] Modification of IOX1 to methyl ester **2** gave an apparently nonselective increase in IC_50_ values. Increasing the length of the ester alkoxy group to four carbons (as in **4**) created apparent selectivity towards a subset of the JmjC KDMs, and in particular the KDM4 subfamily. Further increasing the length of the alkoxy-group to eight carbons (as in **5**) narrowed the observed inhibitory activity to the KDM4 subfamily; specifically, KDM4C was the most potently inhibited enzyme.

**Table 3:** In vitro selectivity of IOX1 (1) and its methyl (2), *n*-butyl (4) and *n*-octyl (5) ester derivatives for JmjC subfamilies

Protein	IC_50_ [μm]^[a]^			
	1	2	4	5
KDM4C	0.6	10.7	5.0	3.9
KDM4E	2.3	12.8	6.3	45.0
KDM2A	1.8	30.1	16.3	>100
KDM3A	0.1	14.5	29.4	>100
KDM5C	19.0	34.9	>100	>100
KDM6B	1.4	10.8	>100	>100
PHD2	33.0	41.1	>100	>100

[a] IC_50_ values derived from in vitro AlphaScreen assays. Data represent the mean of *n*=4 replicates (Figure S8 in the Supporting Information).

The apparent relative selectivity of **5** for the KDM4 subfamily, at least compared with the parent IOX1 (**1**), might be due to differences in the active sites of the JmjC proteins; crystallographic evidence implies that the active site opening of the KDM4 demethylases is larger than that of other JmjC subfamilies, and in particular compared with the narrow binding pocket of the PHD family of hydroxylases.[28–30] This initial characterisation suggests that an appropriate substitution of the IOX1 C-5 position could enable the generation of potent and selective JmjC KDM inhibitors that are active in cells.

In conclusion, we have shown that C-5 ester derivatives of IOX1 can retain JmjC KDM inhibitory activity. Of the tested esters, *n-*octyl derivative **5** was the most potent in vitro against KDM4C. In cells, ester **5** was the least cytotoxic of the tested compounds and the most potent inhibitor of H3K9me3 demethylation (EC_50_=3.8 μm). This is likely to be, at least in part, due to improved cell permeability of **5** compared with that of **1**, as detected in an intracellular delivery assay. Interestingly, it seems that **5** is not, at least efficiently, hydrolysed in HeLa cells, though esterases are known to be present and there are reported examples of short-chain ester hydrolysis.[32, 33] Thus, it seems likely that at least some of the cellular activity of **5** results from inhibition by the intact ester form.

Docking studies based on crystallographic analysis with IOX1 support the viability of *n*-octyl ester **5** binding KDM4, with the alkyl group occupying part of a region leading to the active site. It is notable that some other histone demethylase and deacetylase inhibitors reported in the literature contain an aliphatic chain, two examples with an *n*-octyl group as in **5**, possibly reflecting a general binding of aliphatic groups in this region.[34–37] Binding energies as calculated by docking simulations were found to correlate reasonably well with the AlphaScreen inhibition results and provide a possible explanation for the increased potency of esters with a long alkyl chain.

An extended AlphaScreen with JmjC KDMs and PHD2 as a prolyl hydroxylase representative indicates that increasing the ester chain length to four carbons improves the selectivity towards JmjC KDMs. Most importantly, a chain length of eight carbons—as in derivative **5**—creates selectivity towards the KDM4 subfamily.

The activity of **5** raises the question as to whether other available ester prodrugs of JmjC inhibitors, such as NOG, 2,4-PCDA, GSK-J4, methylstat and 2-hydroxyglutarate, could also be active in their ester forms, and whether systematic ester derivatisation could lead to increased cellular potencies.[19, 20, 34, 38, 39] It is important to note, however, that the results with different 2OG oxygenases reveal that ester derivatisation of IOX1, and possibly of other broad-spectrum KDM inhibitors including the aforementioned compounds, may confer selectivity not apparent in the parent inhibitor.

We hope **5** will find use as a starting point for the development of new JmjC inhibitors as well as a cell-permeable tool compound in studies investigating the role of JmjC histone demethylases as therapeutic targets.

## Experimental Section

Experimental details of the synthesis and characterisation, in vitro assays and cell-based studies, as well as supplementary figures, are given in the Supporting Information available via http://dx.doi.org/10.1002/cmdc.201300428.

## Supporting Information

As a service to our authors and readers, this journal provides supporting information supplied by the authors. Such materials are peer reviewed and may be re-organized for online delivery, but are not copy-edited or typeset. Technical support issues arising from supporting information (other than missing files) should be addressed to the authors.

miscellaneous_information
