# Supplementary material for: A Cell-Permeable Ester Derivative of the JmjC Histone Demethylase Inhibitor IOX1
Source: ChemMedChem. 2014 Feb 6;9(3):566–71. doi: 10.1002/cmdc.201300428 (PMC4503230; doi:10.1002/cmdc.201300428)
Supplement: Supplementary file 1 — miscellaneous_information [file cmdc0009-0566-sd1.pdf]

CHEMMEDCHEM
Chemistry & Drug Discovery

Supporting Information

© Copyright Wiley-VCH Verlag GmbH & Co. KGaA, 69451 Weinheim, 2014

A Cell-Permeable Ester Derivative of the JmjC Histone Demethylase Inhibitor IOX1

Rachel Schiller,[a] Giuseppe Scozzafava,[b] Anthony Tumber,[b] James R. Wickens,[a] Jacob T. Bush,[a] Ganesha Rai,[c] Clarisse Lejeune,[a] Hwanho Choi,[a] Tzu-Lan Yeh,[a] Mun Chiang Chan,[a] Bryan T. Mott,[c] James S. O. McCullagh,[a] David J. Maloney,[c] Christopher J. Schofield,*[a] and Akane Kawamura*[a, d]

cmdc_201300428_sm_miscellaneous_information.pdf

Supporting Information

Experimental Methods

Chemistry

General Experimental

Reagents and solvents were, unless otherwise stated, from Sigma Aldrich, Alfa Aesar or Acros and were used as received. Solvents were removed under reduced pressure using a Buchi™ rotary evaporator. Water was purified using an Elix® UV-10 system.

Analytical thin layer chromatography (TLC) was carried out on Merck silica gel 60 F254 aluminium supported thin layer chromatography sheets. Visualisation was by absorption of UV light (λ_{max} 254 nm).

Flash Column chromatography was carried out using a Biotage SP1 automated flash column chromatography platform, eluting with indicated solvents under a positive pressure of compressed air.

Melting points were determined using a Leica Galen III hot stage melting point apparatus and microscope.

Infrared spectra were obtained as a thin film on sodium chloride discs. The spectra were recorded on a Bruker Tensor 27 spectrometer and a representative number of absorption maxima are reported in wavenumbers (cm^{-1}).

^{1}H NMR spectra were recorded on Bruker DPX400 (400 MHz) using deuterochloroform or DMSO-d_6 as a reference for internal deuterium lock. The chemical shifts data are given as δ in units of parts per million (ppm) relative to tetramethylsilane (TMS) where δ(TMS) = 0.00 ppm. The multiplicity of each signal is indicated by: s (singlet); app. br s (apparent broad singlet); d (doublet); t (triplet); q (quartet); dd (doublet of doublets); ddd (doublet of doublet of doublets); td (triplet of doublets) or m (multiplet). The number of protons (n) for a given resonance signal is indicated by nH. Coupling constants (*J*) are expressed in Hz and are recorded to the nearest 0.5 Hz. Coupled proton coupling constants (*J*) are averaged and reported to the nearest 0.5 Hz.

^{13}C NMR spectra were recorded on a Bruker AV400 (101 MHz) spectrometer using the PENDANT or DEPT Q pulse sequences with broadband proton decoupling and internal deuterium lock. The chemical shift data for each signal are given as δ in units of parts per million (ppm) relative to tetramethylsilane (TMS) where δC (TMS) = 0.00 ppm. ^{1}H and ^{13}C spectra were assigned using 2D NMR experiments including COSY and HSQC.

Mass spectra were acquired on Agilent technologies 6120 quadruple LC/MS spectrometer using electrospray ionisation, operating in positive or negative mode, from sample solutions in MeOH. *m/z* values are reported in Daltons and followed by their percentage abundance in parentheses. High resolution mass spectra (HRMS) were recorded using Bruker MicroTOF internally calibrated with polyalanine.

Synthetic Procedures and Characterisation for Compounds

^{1}H NMR and HRMS spectra are presented in Figure S9.

8-Hydroxyquinoline-5-carboxylic acid (1)

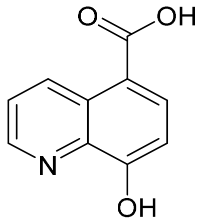

Acrolein (550 mg, 0.65 mL, 9.81 mmol, 1.5 eq.) was added dropwise over 30 min to a solution of 3-amino-4-hydroxybenzoic acid (1.0 g, 6.54 mmol, 1.0 eq.) in 6 N HCl. The reaction mixture was refluxed at 100 °C for 2 h in a 50 mL round bottom flask fitted with a jacketed water condenser. Upon completion of the reaction based on TLC analysis, the reaction mixture was allowed to cool to room temperature and the pH was adjusted to pH 9 with aqueous ammonia. The mixture was then filtered and the filtrate was acidified to pH 4-5 with 10% aqueous acetic acid. The resulting precipitate was obtained by filtration, washed with water (10 mL) and dried in vacuo to yield the desired product **1** as a brown powder (642 mg, 3.40 mmol, 52%). R_f = 0.35 (CH_2Cl_2 / MeOH (3:1)); m.p. 272-273 °C (decomposition); ^{1}H NMR (400 MHz, DMSO-d_6) δ = 7.13 (1 H, d, *J* = 8.0 Hz, C(Ar)*H*), 7.70 (1 H, dd, *J* = 8.5, 4.0 Hz, C(Ar)*H*), 8.25 (1 H, d, *J* = 8.0 Hz, C(Ar)*H*), 8.92 (1 H, dd, *J* = 4.0, 1.5 Hz, C(Ar)*H*), 9.47 (1 H, dd, *J* = 8.5, 1.5 Hz, C(Ar)*H*) ppm; ^{13}C-NMR (101 MHz, DMSO-d_6) δ = 111.0 (*C*(Ar)), 117.2 (*C*(Ar)), 124.1 (*C*(Ar)), 128.9 (*C*(Ar)), 134.4 (*C*(Ar)), 135.3 (*C*(Ar)), 139.1 (*C*(Ar)), 149.0 (*C*(Ar)), 158.7 (*C*(Ar)), 168.5 (*C*OO) ppm; FT-IR v_{max}: 3210 (OH), 1684 (C=O) cm^{-1}; LRMS *m*/*z* (ESI$^+$) 191 ([M+2H] $^{2+}$, 100%); HRMS (ESI$^-$) $C_{10}H_6NO_3$ ([M-H]$^-$) requires: 188.0353; found: 188.0351. These data are consistent with those previously reported.[1]

Methyl 8-hydroxyquinolone-5-carboxylate (2)

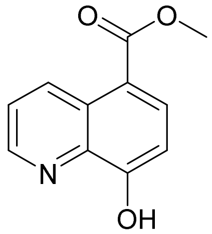

To a mixture of 5-bromoquinolin-8-ol (4 g, 17.85 mmol), triphenylphosphine (9.37 g, 35.7 mmol) and 2-(trimethylsilyl)ethanol (3.82 mL, 26.8 mmol) in tetrahydrofuran (THF) (40 mL) and toluene (40 mL) was slowly added diisopropyl azodicarboxylate (DIAD) (7.40 mL, 35.7 mmol). The reaction mixture was then stirred at room temperature for 24 h. After completion of the reaction, the reaction mixture was concentrated and purified using a Biotage Flash system eluting with 25% EtOAc in hexanes to afford 5.1 g (5.1 g, 15.73 mmol, 88%) of 5-bromo-8-(2-(trimethylsilyl)ethoxy)quinoline as a colorless oil. To a degassed solution of 5-bromo-8-(2-(trimethylsilyl)ethoxy)quinoline (5.1 g, 15.73 mmol) in methanol (40 mL) and DMSO (40 mL) was added palladium(II)acetate (0.353 g, 1.57 mmol), 1,3-bis(diphenylphosphino)propane (1.30 g, 3.15 mmol) and Et_3N (8.8 mL, 62.9 mmol). The reaction mixture was bubbled with carbon monoxide for 5 minutes then stirred under a carbon monoxide atmosphere (balloon pressure) for 16 h. The crude reaction mixture was then extracted twice with CH_2Cl_2. The organic layer was washed successively with water, ammonium chloride and brine. The crude product

obtained upon concentration was purified using a Biotage Flash system eluting with 25% EtOAc in hexanes to afford colourless oil (4.6 g, 22.64 mmol, 96%). 1.3 g of the pure product was dissolved in 12 mL of CH_2Cl_2/CF_3CO_2H (1:1 by volume) mixture and heated in a Biotage microwave reactor at 100 °C for 15 minutes to give **2**. The solvent was evaporated off and the crude product was purified by preparative HPLC. ^{1}H NMR (400 MHz, DMSO-*d6*) δ = 3.89 (3 H, s, C*H*$_3$), 7.14 (1 H, d, *J* = 8.0 Hz, C(Ar)*H*), 7.72 (1 H, ddd, *J* = 9.0, 4.0, 0.5 Hz, C(Ar)*H*), 8.25 (1 H, dd, *J* = 8.0, 0.5 Hz, C(Ar)*H*), 8.96 - 8.89 (1 H, m, C(Ar)*H*), 9.36 (1 H, ddd, *J* = 9.0, 2.0, 0.5 Hz, C(Ar)*H*), 10.79 (1 H, s, O*H*) ppm; ^{13}C NMR (101 MHz, DMSO-*d*$_6$) δ = 51.8 (*C*H$_3$), 110.3 (*C*(Ar)), 115.41 (*C*(Ar)), 123.5 (*C*(Ar)), 127.8 (*C*(Ar)), 133.4 (*C*(Ar)), 134.1 (*C*(Ar)), 138.1 (*C*(Ar)), 148.3 (*C*(Ar)), 158.2 (*C*(Ar)), 166.1 (*C*OO) ppm; LRMS *m*/z (ESI$^+$) 204 ([M+H]$^+$, 100%);HRMS *m*/z (ESI$^+$) calculated for $C_{11}H_{10}NO_3^+$, [M+H]$^+$ = 204.0655; found [M+H]$^+$ =204.0659.

Ethyl 8-hydroxyquinoline-5-carboxylate (3)

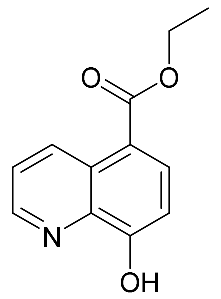

8-Hydroxyquinoline-5-carboxylic acid (100 mg, 0.53 mmol) was heated at reflux for 2 days in EtOH containing 3 drops of concentrated H_2SO_4. The cooled solution was evaporated in vacuo, the mixture was dissolved in methanol and the resulting crude purified using semi-preparative reverse-phase HPLC, performed on a WATERS sunfire C18 column (150 mm x 10 mm, 5 μm). Separation was achieved using a linear gradient of solvent A (water + 0.1% CF_3CO_2H) and solvent B (acetonitrile + 0.1% CF_3CO_2H), eluting at a flow rate of 5 mL/min and monitoring at 254 nm: 2% B over 2 min and 2% B to 90% B over 18 min, to give 15 mg of compound **3** as a yellow solid (15 mg, 0.07 mmol, 13%). R_f = 0.35 (CH_2Cl_2 / MeOH (9:1)); m.p. 196-203 °C**;** ^{1}H NMR (500 MHz, CD_3OD) δ = 1.49 (3H, t, *J* = 7.1 Hz, C*H*$_3$), 4.52 (2 H, q, *J* = 7.1 Hz, C*H*$_2$), 7.44 (1 H, d, *J* = 8.4 Hz, C(Ar)*H*), 8.13 (1 H, dd, *J* = 8.9, 5.0 Hz, C(Ar)*H*), 8.58 (1 H, d, *J* = 8.4 Hz, C(Ar)*H*), 9.09 (1 H, dd, *J* = 5.0, 1.4 Hz, C(Ar)*H*), 10.14 (1 H, dd, *J* = 8.9, 1.4 Hz, C(Ar)*H*) ppm; ^{13}C NMR (500 MHz, CD_3OD) δ = 14.7 (*C*H$_3$), 61.8 (*C*H$_2$), 110.6 (*C*(Ar)), 124.4 (*C*(Ar)), 129.7 (*C*(Ar)), 131.1 (*C*(Ar)), 134.7 (*C*(Ar)), 136.1 (*C*(Ar)), 139.7 (*C*(Ar)), 149.4 (*C*(Ar)), 159.2 (*C*(Ar)), 167.8 (*C*OO) ppm; FT-IR v_{max}: 2924 (OH), 1705 (C=O) cm^{-1}; HRMS *m*/z (ESI$^+$) calculated for $C_{12}H_{12}NO_3^+$, [M+H]$^+$ = 218.0812; found [M+H]$^+$ = 218.0805.

***n*-Butyl 8-hydroxyquinoline-5-carboxylate (4)**

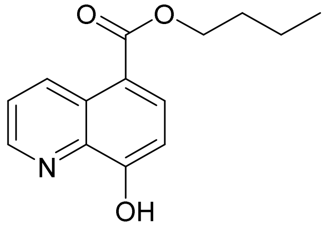

A solution of **1** (100 mg, 0.53 mmol) in *n*-butanol (10 mL) and one drop of concentrated H_2SO_4 was refluxed at 120 °C for 21 h in a 50 mL round bottom flask fitted with a jacketed water condenser. Upon completion of the reaction, based on TLC analysis, the reaction mixture was neutralised with 20% aqueous NaOH and the excess *n*-butanol was removed in vacuo. The residue was dissolved in ethylacetate (EtOAc, 10 mL), washed with sat. $NaHCO_3$ (aq) (10 mL) and water (2 x 10 mL)), dried over

Na_2SO_4 and concentrated in vacuo. The resulting brown gum was purified by flash silica gel chromatography (gradient elution 12-50% CH_2Cl_2 in cyclohexane (*c*Hex)) to give **4** as a sand-yellow solid (33 mg, 0.14 mmol, 25%). R_f = 0.30 (EtOAc / *c*Hex (1:1)); m.p. 76.5-77.7 °C; ^{1}H NMR (400 MHz, $CDCl_3$) δ = 1.01 (3 H, t, *J* = 7.5 Hz, CH_3), 1.39 - 1.63 (2 H, m, CH_2CH_3), 1.68 - 2.02 (2 H, m, $CH_2CH_2CH_3$), 4.38 (2 H, t, *J* = 7.0 Hz, $CH_2CH_2CH_2CH_3$), 7.17 (1 H, d, *J* = 8.0 Hz, C(Ar)*H*), 7.59 (1 H, dd, *J* = 9.0 , 4.0 Hz, C(Ar)*H*), 8.36 (1 H, d, *J* = 8.0 Hz, C(Ar)*H*), 8.81 (1 H, dd, *J* = 4.0, 1.5 Hz, C(Ar)*H*), 9.52 (1 H, dd, *J* = 9.0, 1.5 Hz, C(Ar)*H*) ppm; ^{13}C NMR (101 MHz, $CDCl_3$) δ = 13.8 (CH_3), 19.4 (CH_2CH_3), 30.9($CH_2CH_2CH_3$), 64.7 (OCH_2), 108.6 (*C*(Ar)), 116.9 (*C*(Ar)), 123.4 (*C*(Ar)), 128.0 (*C*(Ar)), 133.7 (*C*(Ar)), 135.4 (*C*(Ar)), 137.9 (*C*(Ar)), 147.8 (*C*(Ar)), 156.5 (*C*(Ar)), 166.4 (*C*OO) ppm; FT-IR ν_{max}: 3200 (OH), 2959-2873 (C-H), 1693 (C=O) cm^{-1}; LRMS *m*/*z* (ESI$^+$) 246 ([M+H]$^+$, 100%); HRMS *m*/*z* (ESI$^+$) calculated for $C_{14}H_{16}NO_3^+$, [M+H]$^+$ = 246.1125; found [M+H]$^+$ = 246.1121.

***n*-Octyl 8-hydroxyquinoline-5-carboxylate (5)**

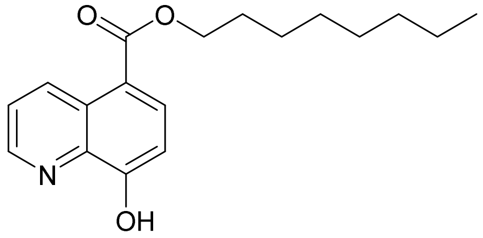

A solution of **1** (100 mg, 0.53 mmol) in *n*-octanol (10 mL) and one drop of concentrated H_2SO_4 was refluxed at 120 °C for 20 h in a 50 mL round bottom flask fitted with a jacketed water condenser. Upon completion of the reaction based on TLC analysis, the reaction mixture was neutralised with 20% aqueous NaOH. Excess *n*-octanol was removed by rotary evaporation at 70 °C and 15 mbar. The residue was further purified by flash silica gel chromatography (eluting with 6 column volumes of *c*Hex followed by gradient elution with 0 to 100% CH_2Cl_2 in *c*Hex) to give **5** as a light-yellow solid (39 mg, 0.13 mmol, 25%). R_f = 0.40 (CH_2Cl_2 / MeOH (19:1)); m.p. 82.0-84.1 °C; ^{1}H NMR (400 MHz, $CDCl_3$) δ = 0.89 (3 H, t, *J* = 6.5 Hz, CH_3), 1.24 - 1.56 (10 H, m, $CH_3(CH_2)_5$), 1.76 - 1.90 (2 H, m, $COOCH_2CH_2$), 4.37 (2 H, t, *J* = 6.5 Hz, $COOCH_2$), 7.18 (1 H, d, *J* = 8.5 Hz, C(Ar)*H*), 7.59 (1 H, dd, *J* = 8.5, 4.0 Hz, C(Ar)*H*), 8.36 (1 H, d, *J* = 8.5 Hz, C(Ar)*H*), 8.81 (1 H, dd, *J* = 4.0, 1.5 Hz, C(Ar)*H*), 9.52 (1 H, dd, *J* = 8.5, 1.5 Hz, C(Ar)*H*) ppm; ^{13}C NMR (101 MHz, $CDCl_3$) δ = 14.1(CH_3), 22.7 (CH_3CH_2), 26.2 ($COO(CH_2)_2CH_2$), 28.8 ($COOCH_2CH_2$), 29.2 ($COO(CH_2)_4CH_2$) , 29.3 ($COO(CH_2)_3CH_2$), 31.8 ($COO(CH_2)_5CH_2$), 65.0 ($COOCH_2$), 108.6 (*C*(Ar)), 116.9 (*C*(Ar)), 123.4 (*C*(Ar)), 128.0 (*C*(Ar)), 133.7 (*C*(Ar)), 135.4 (*C*(Ar)), 137.9 (*C*(Ar)), 147.8 (*C*(Ar)), 156.5 (*C*(Ar)), 166.4 (*C*OO) ppm; FT-IR ν_{max}: 3297 (OH), 2857-2955 (C-H), 1702 (C=O) cm^{-1}; LRMS *m*/*z* (ESI$^+$) 302([M+H]$^+$, 100%); HRMS *m*/*z* (ESI$^+$) calculated for $C_{18}H_{24}NO_3^+$, [M+H]$^+$ = 302.1651; found [M+H]$^+$ = 302.1744.

Methyl 8-acetoxyquinolone-5-carboxylate (6)

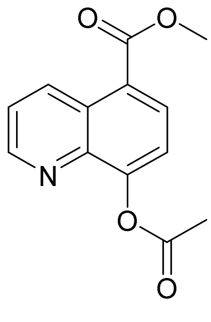

A mixture of **2** (0.1 g, 0.492 mmol), 4-dimethylaminopyridine (DMAP) (0.012 g, 0.098 mmol) and acetic anhydride (0.232 mL, 2.461 mmol) in CH_2Cl_2 (3 mL) was refluxed for 1 h. The reaction mixture was concentrated and purified using a Biotage Flash system eluting with 20% EtOAc in hexanes to afford compound **6** as a white solid. ^{1}H NMR (400 MHz, DMSO-d_6) δ = 2.44 (3 H, s, C*H*$_3$COO), 3.96 (3 H, s, C*H*$_3$OCO), 7.74 (1 H, dd, *J* = 9.0, 4.0 Hz, C(Ar)*H*), 7.66 (1 H, d, *J* = 8.0 Hz, C(Ar)*H*), 8.29 (1 H, d, *J* = 8.0 Hz, C(Ar)*H*), 8.99 (1 H, dd, *J* = 4.0, 1.5 Hz, C(Ar)*H*), 9.24 (1 H, dd, *J* = 9.0, 1.5 Hz, C(Ar)*H*) ppm; LRMS *m*/z (ESI$^+$) 246 ([M+H]$^+$, 100%); HRMS *m*/z (ESI$^+$) calculated for $C_{13}H_{11}NO_4Na^+$, [M+H]$^+$ =268.0580; found [M+H]$^+$ =268.0574.

(Pivaloyloxy)methyl-8-((pivaloyloxy)methoxy)quinoline-5-carboxylate (7)

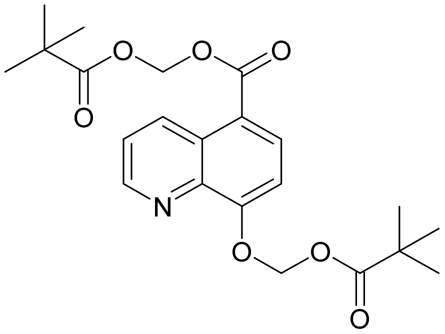

To a mixture of **1** (100 mg, 0.53 mmol, 1.0 eq.) and chloromethyl pivalate (77 µL, 0.53 mmol, 1.0 eq.) in a mixture of acetone / dimethylformamide (1:1, 10 mL) was added Et_3N (54 mg, 0.53 mmol, 1.0 eq.). The mixture was refluxed at 50 °C for 3 h, whereupon a small amount of precipitate was formed. The precipitate was filtered and washed with acetone (20 mL). The filtrate was evaporated and the residue was partitioned between 5% w/w aqueous $NaHCO_3$ (30 mL) and EtOAc (20 mL). The organic phase was collected, dried over $MgSO_4$ and concentrated in vacuo. The residue was further purified by flash silica gel chromatography (gradient elution of 0 to 40% EtOAc in *c*Hex (15 column volumes) followed by 5 column volumes of 40% EtOAc in *c*Hex) to give **7** as a yellow oil (52 mg, 0.13 mmol, 24%). R_f = 0.45 (*c*Hex / EtOAc (3:2)); ^{1}H NMR (400 MHz, $CDCl_3$) δ = 1.22 (9 H, s, (C*H*$_3$)$_3$), 1.25 (9 H, s, (C*H*$_3$)$_3$), 6.07 (2 H, s, OC*H*$_2$O), 6.16 (2 H, s, OC*H*$_2$O), 7.32 (1 H, d, *J* = 8.0 Hz, C(Ar)*H*), 7.60 (1 H, dd, *J* = 9.0, 4.0 Hz, C(Ar)*H*), 8.38 (1 H, d, *J* = 8.0 Hz, C(Ar)*H*), 9.01 (1 H, dd, *J* = 4.0, 1.5 Hz, C(Ar)*H*), 9.47 (1 H, dd, *J* = 9.0, 1.5 Hz, C(Ar)*H*) ppm; ^{13}C NMR (101 MHz, DMSO-d_6) δ = 26.5 (2x(*C*H$_3$)$_3$), 38.3 (*C*(CH$_3$)$_3$), 38.4 (*C*(CH$_3$)$_3$), 80.0 (O*C*H$_2$O), 85.4 (O*C*H$_2$O), 111.3 (*C*(Ar)), 118.4 (*C*(Ar)), 123.7 (*C*(Ar)), 127.9 (*C*(Ar)), 132.7 (*C*(Ar)), 133.4 (*C*(Ar)), 139.5 (*C*(Ar)), 150.0 (*C*(Ar)), 156.6 (*C*(Ar)), 164.0 (C(5)*C*OO), 176.3 (*C*OC(CH$_3$)$_3$), 176.5 (*C*OC(CH$_3$)$_3$) ppm; FT-IR ν_{max}: 2975 (C-H), 1725 (C=O) cm^{-1}; LRMS *m*/z (ESI$^+$) 418 ([M+H]$^+$, 100%); HRMS *m*/z (ESI$^+$) calculated for $C_{22}H_{27}NO_7^+$, [M+H]$^+$ = 417.1787; found [M+H]$^+$ = 417.1801.

Biological Procedures

Cell Culture

The human cervical carcinoma HeLa cell line was from the American Type Cultures Collection (ATCC, Manassas, VA) and cultured in Dulbecco's modified Eagle medium (DMEM; Invitrogen Gibco Cell Culture Products, Carlsbad, CA) supplemented with 10% FCS (Invitrogen), 1% Glutamax (Invitrogen)

and 1% penicillin-streptomycin (Lonza). Cultures were kept at 37 °C in a humidified atmosphere of 95% air and 5% CO_2. Cells were detached from the culture flask with a solution of 0.05% trypsin and 0.02% Ethylenediaminetetraacetic acid (EDTA; Sigma) and then washed and suspended in complete culture medium with 10% FCS.

Viability analysis in HeLa cells

Antiproliferative activities of compounds were determined by the MTT (3-[4,5-dimethylthiazol-2-yl]-2,5 diphenyl tetrazolium bromide) assay. HeLa cells were seeded into 96-well plates (2000 cells/well) and cultured at 37 °C for 24 h to achieve 70% confluency. Subsequently, the medium was replaced with DMEM medium containing the tested compounds in different concentrations of 1-300 μM in 1% DMSO. Staurosporine in 0.03-10 μM final concentration was used as a control for cytotoxicity. After 24 h of treatment, the medium was replaced with CellTiter 96® Aqueous One Solution Reagent (Promega) and incubated for 4 h. CC_{50} values were calculated in Prism 6 software after normalisation against corresponding 1% DMSO treated cells and 1% DMSO in media (no cells) controls.

Cellular Demethylase Assay

A protocol adapted from that of King et al., 2010 was used for investigation of cell KDM inhibition activity.[2] HeLa cells were maintained in DMEM media supplemented with 10% FBS, penicillin and streptomycin. Cells were transiently transfected with either Flag-tagged KDM4A or the H188A catalytic inactive variant of KDM4A using Fugene HD. Inhibition studies were initiated 24 h after cellular transfection and compounds were added to a final concentration of 1-300 μM in 1% DMSO. H3K9me3 levels were measured in cells following 24 h incubation with compound. All cells were stained with an anti-Flag mouse monoclonal antibody (Sigma F1804), rabbit anti-H3K9me3 (Abcam Ab8898) and DAPI (Sigma D9564) for DNA. Alexa Fluor® 488 antibody (Life technologies A-21121) and Alexa Fluor® 568 antibody (Life technologies A-11011) were used to fluorescently label the Flag and H3K9me3 primary antibodies. Image acquisition was conducted using Operetta High Content Imaging System (PerkinElmer) and image analysis was performed with Harmony High Content Imaging and Analysis Software (PerkinElmer). The nuclei were automatically identified by DAPI staining using the default parameters. Cells expressing low amounts of the exogenous demethylase, where Flag-tag staining was dim, were omitted from analysis by setting a minimum threshold. EC_{50} values were calculated in Prism 6 after normalisation by setting DMSO treated KDM4A transfected cells to 100% demethylase activity and the catalytic inactive KDM4A variant transfected cells to 0% activity.

AlphaScreen Assay

A protocol adapted from that of Kawamura *et al.*, 2010 was used.[3] All reagents were diluted in 50 mM HEPES, 0.1% BSA, pH 7.5 supplemented with 0.01% Tween20 and allowed to equilibrate to room temperature prior to addition to plates. Catalytic turnover assays were run in 10 µL volumes in low-volume 384-well plates (ProxiPlateTM-384 Plus, PerkinElmer, USA) at RT. The reaction consisted of enzyme (5 nM), biotinylated substrate peptide (30 nM), Fe(II) (1 µM), ascorbate (100 µM), 2OG (10 µM) and run at RT. For PHD2, the reaction consisted of enzyme (5 nM), biotinylated substrate peptide (60nM), Fe(II) (20 μM), ascorbate (200 μM), 2OG (2 μM) and run at RT.[4] EDTA was used to quench the reaction (5 µL), AlphaScreen donor (Streptavidin-conjugated) and acceptor (Protein A-conjugated) beads preincubated with peptide product antibodies were added (5 µL). Plates were foil-sealed to protect from light, incubated at room temperature for 60 minutes and read on a PHERAstar FS plate reader (BMG Labtech, Germany) using an AlphaScreen 680 excitation/570 emission filter set. The final bead concentration in 20 µL reaction was 20 µg/mL. IC_{50} values were calculated in Prism 6 after normalisation against corresponding DMSO controls.

Test of Compound Stability in the AlphaScreen Buffer

3 mM of the tested compounds were incubated in a solution composed of AlphaScreen buffer (50 mM HEPES pH 7.5 supplemented with 0.1% BSA, 0.01% Tween20, Fe(II) (10 µM), ascorbate (100µM) and 2OG (10 µM)) in room temperature. Samples were taken for LCMS analysis after 0, 1, 2 and 24 h of incubation. LCMS retention times (t_r) are quoted to the nearest 0.1 min. LCMS was performed on a WATERS Sunfire equipped with a C18 column (150 mm x 4.6 mm, 5 µm) using a linear gradient of solvent A (water + 0.1% CF_3CO_2H) and solvent B (acetonitrile + 0.1% CF_3CO_2H), eluting at a flow rate of 1 mL/min and monitoring at 254 nm: 2% B over 2 min, 2% B to 100% B over 16 min and 100% B over 2 min.

Intracellular Delivery Assay

The protocol used was adapted from that of Kruidenier *et al*., 2010.[5] HeLa cells were dosed with 200 µM IOX1 **1**, *n*-octyl ester **5** or DMSO. After 24 h of incubation the media was aspirated and the cells were washed with PBS. The number of cells in each sample was determined. Extraction of the compounds from the cells was performed by lysis using 80% aqueous methanol. All samples were shaken for 10 min on a vortex mixer and then centrifuged for 10 min at 15000 rpm. The supernatant was removed and the methanol solution was evaporated using a SpeedVac machine. Samples were dissolved with water in proportion to the cell count and an aliquot of the resulting supernatant was mixed with caffeine at 1000 ng/mL as the internal standard. Samples were analysed by reverse phase LC MS/MS using a heat assisted electrospray interface in positive ion mode. The instrument used was a Waters Quattro Micro triple quadrupole mass spectrometer coupled to liquid chromatrography CTC PAL and Waters Acquiy UPLC. The column was Gemini C18 HPLC with 3.0 mm internal diameter (Phenomenex). Nominal MRM (Multiple Reaction Monitoring) transitions for analytes were 190.0 to 146.0 for IOX1 **1**, 302.2 to 190.0 for ester **5** and 195.0 to 138.1 for Caffeine. MRM methods were ran over a 5 minutes gradient running from 1% ACN + 0.5% acetic acid (aq) to 90% ACN + 10% formic acid (aq), held for 2 minutes and returned to the starting conditions over 0.1 minutes and remained at the starting conditions for 3 minutes. Samples were assayed against calibration standard curve over the range of 0 to 200 µM prepared with diluted cell lysate of cells dosed with DMSO. Quantitative analysis was made by measuring the peak area of the compounds, normalised using caffeine, followed by linear regression analysis to calculate the number of moles per cell (Figure S3).

Cell culture and immunoblotting for HIF hydroxylase inhibition assay

Cells (Hep3B, RCC4 and HeLa) were cultured in DMEM each supplemented with 10% fetal calf serum, 2 mM L-glutamine, 50 units/ml of penicillin, and 50 µg/ml of streptomycin. Cells were treated with compounds for 5-6 h and harvested for immunoblotting as previously described.[6] All compounds (except DMOG) were dissolved in DMSO and added directly to culture medium at final DMSO concentrations of ≤2%. DMOG (dimethyloxalylglycine, dissolved in water) and FG2216 were used as positive controls. MG132 treatment was for 4 h. Antibodies to HIF-1α and β-actin/HRP were from BD Transduction Laboratories (clone-54) and Abcam (clone, AC-15) respectively and an antibody to hydroxyAsn803 (HyAsn803) was a kind gift from Dr Myung Kyu Lee (BioNanotechnology Research Centre, KRIBB, Republic of Korea).

Docking experiments

For protein-ligand docking simulations, an X-ray crystal structure of KDM4A in complex with IOX1 (PDB ID - 3NJY) was employed.[2] Attention was paid to the assignment of protonation states for Asp, Glu, His and Lys residues. Atomic charges were then assigned to the all-atom model of KDM4A through the restrained electrostatic potential (RESP) methods using the AMBER program. Due to the need for electronic charge redistribution resulting from the ferrous ion and its ligands, quantum mechanical calculation was performed at the PBE0/6-31** level of theory with geometry optimization for a simplified model of ferrous ion in complex with the amino acid residues and the posed ligand IOX1.[7,8]

The calculated RESP atomic charges of the active site ferrous ion and its ligand atoms in the KDM4A-IOX1 complex model were as follows (in e): Fe: +1.087, His188 NE: -0.292, His276 NE: -0.213, Glu190 OD: -0.630, IOX1 NAC: -0.592, IOX1 OAN: -0.587. This calculation indicates that the atomic charge of the ferrous ion decreases from +2.000 to +1.087 e upon the formation of the inhibitor complex. The assigned RESP charges of the nitrogen atoms of the two histidine residues are -0.292 and -0.213 respectively; the oxygen of Glu, the nitrogen of IOX1 and the phenolic oxygen of IOX1 are assigned charges -0.630, -0.592 and -0.587 respectively, similar to the values calculated in the absence of the ferrous ion. These changes reflect the redistribution of charges between the ferrous ion and its ligand atoms during the formation of the metal complex.

The RESP partial charges for the atoms detailed above were recalculated for each of the IOX1 ester derivatives based on the calculation done for IOX1. For the docking simulations of each of the IOX1 ester derivatives, the empirical AutoDock scoring function was used, improved by the implementation of a new solvation model for each compound.[9] The modified scoring function has the following form:

$$\Delta G_{bind}^{aq} = W_{vdW}\sum_{i=1}\sum_{j>i}\left(\frac{A_{ij}}{r_{ij}^{12}}-\frac{B_{ij}}{r_{ij}^{6}}\right)+W_{hbond}\sum_{i=1}\sum_{j>i}E(t)\left(\frac{C_{ij}}{r_{ij}^{12}}-\frac{D_{ij}}{r_{ij}^{10}}\right)$$

$$+W_{elec}\sum_{i=1}\sum_{j>i}\frac{q_i q_j}{\varepsilon(r_{ij})r_{ij}}+W_{tor}N_{tor}+\Delta G_{sol} = \sum_{i}^{atoms}\left\{S_i\left(O_i^{\max}-\sum_{j}^{i\neq j}V_j e^{-\frac{r_{ij}^2}{2\sigma^2}}\right)+P_i\sum_{j}^{i\neq j}V_j e^{-\frac{r_{ij}^2}{2\sigma^2}}\right\}$$

Where W_{vdW}, W_{hbond}, W_{elec}, W_{tor}, and W_{sol} are the weighting factors of van der Waals, hydrogen bond, electrostatic interactions, torsional term and desolvation energy of the inhibitors, respectively. r_{ij} represents the interatomic distance and A_{ij}, B_{ij}, C_{ij}, and D_{ij} are related to the depths of the potential energy well and the equilibrium separations between two atoms. The hydrogen bond term has an additional weighting factor, $E(t)$, representing the angle-dependent directionality. A cubic equation approach was applied to obtain the dielectric constant required to compute the interatomic electrostatic interactions between KDM4A and **5**. In entropic terms, N_{tor} is the number of sp^3 bonds in the ligand. In desolvation terms, S_i , P_i and V_i are the solvation parameters, self-solvation parameter and the fragmental volume of atom *i*, respectively, while Occ_i^{max} is the maximum atomic occupancy.[10,11]

Supplementary Figures

**Figure S1**	HeLa Cell viability analysis after dosing with IOX1 ester derivatives
**Figure S2**	Analysis of H3K9me3 demethylation inhibition using immunofluorescence assays
**Figure S3**	Normalised calibration curves used to determine the intracellular levels of compounds by linear regression
**Figure S4**	Effects of IOX1 and IOX1 ester derivatives on HIF in human cell lines
**Figure S5**	KDM4C peptide turnover assayed by AlphaScreen
**Figure S6**	Measurements of the stability of IOX1 **1** and *n*-octyl ester **5** in the AlphaScreen buffer by LCMS
**Figure S7**	Surface view of modelled *n*-decyl IOX1 ester derivative in the KDM4A active site
**Figure S8**	*In vitro* selectivity for JmjC subfamilies
**Figure S9**	^{1}H NMR and HRMS for compounds **1** through **7**
**Table S1**	The Gibbs free energy for binding of IOX1 1 and its ester derivatives to KDM4A as calculated by docking simulations

a)

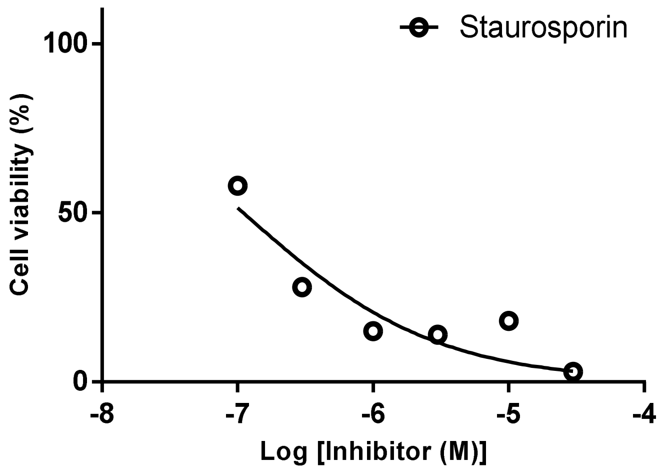

b)

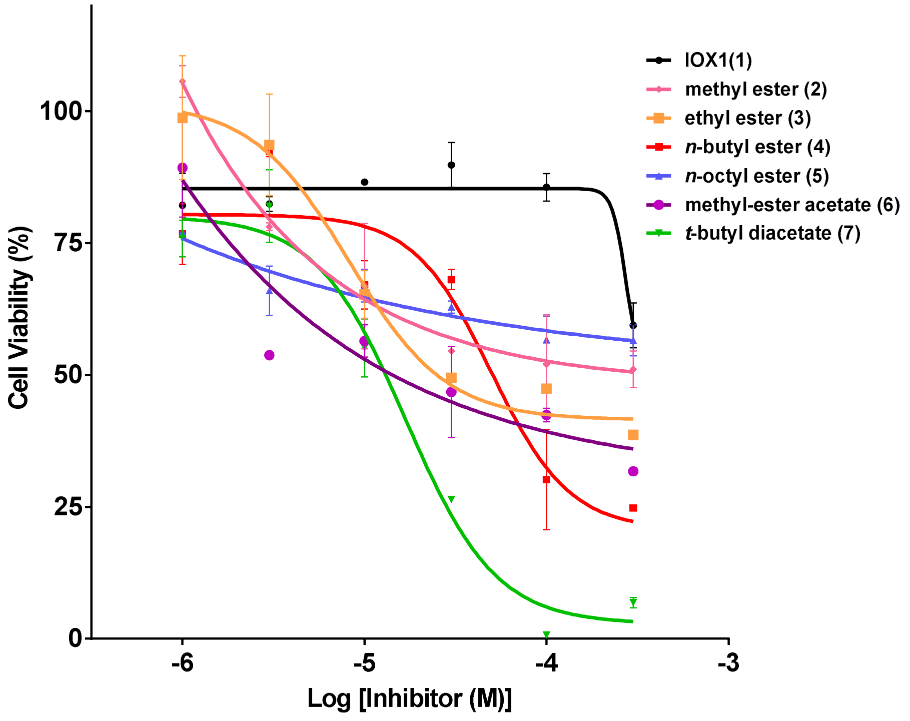

Figure S1- HeLa cell viability analysed after dosing with IOX1 ester derivatives. a) Staurosporine control for cytotoxicity at varied concentrations (0.03 - 10 μM); b) Cytotoxicity measurements of IOX1 **1** and its ester derivatives at varied concentrations (1 - 300 μM) as described in the biological procedures section. Values are mean ± SD, n=3.

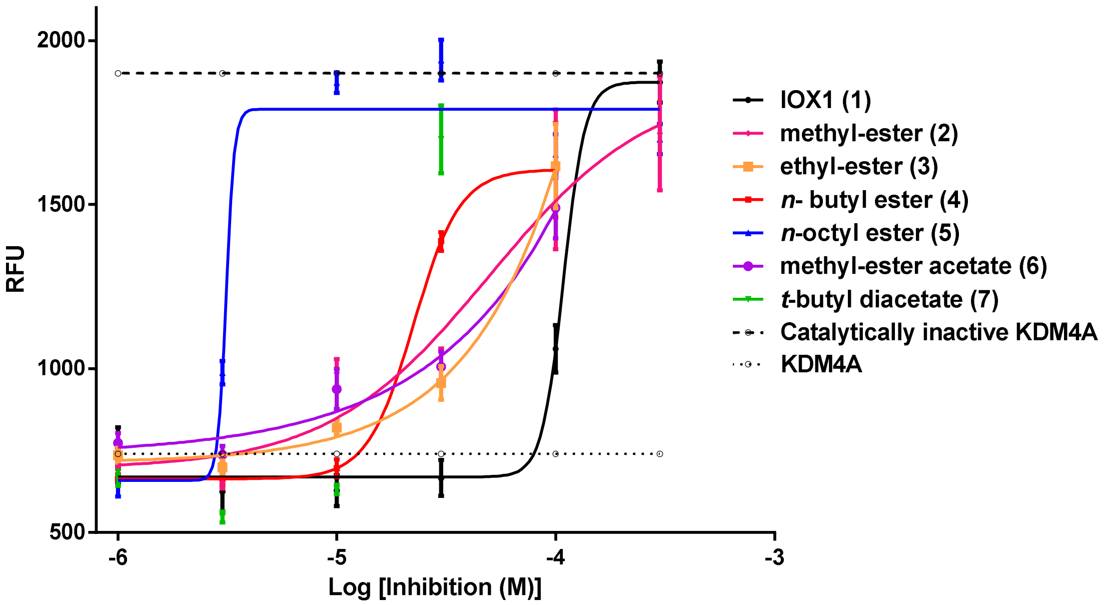

Figure S2 - Analysis of H3K9me3 demethylation inhibition using immunofluorescence assays. Indirect immunofluorescence with anti-Flag (Sigma F1804), anti-H3K9me3 (Abcam Ab8898), and DAPI (Sigma D9564) staining in HeLa cells overexpressing Flag-tagged KDM4A or the H188A catalytic inactive variant of KDM4A. Quantitation of H3K9me3 levels are reflected in Relative Fluorescence Units (RFU). Data outside the quantification range was removed. EC_{50} values are listed in Table 1. Values are mean ± SE. The procedure for this assay is described in the biological procedures section.

a)

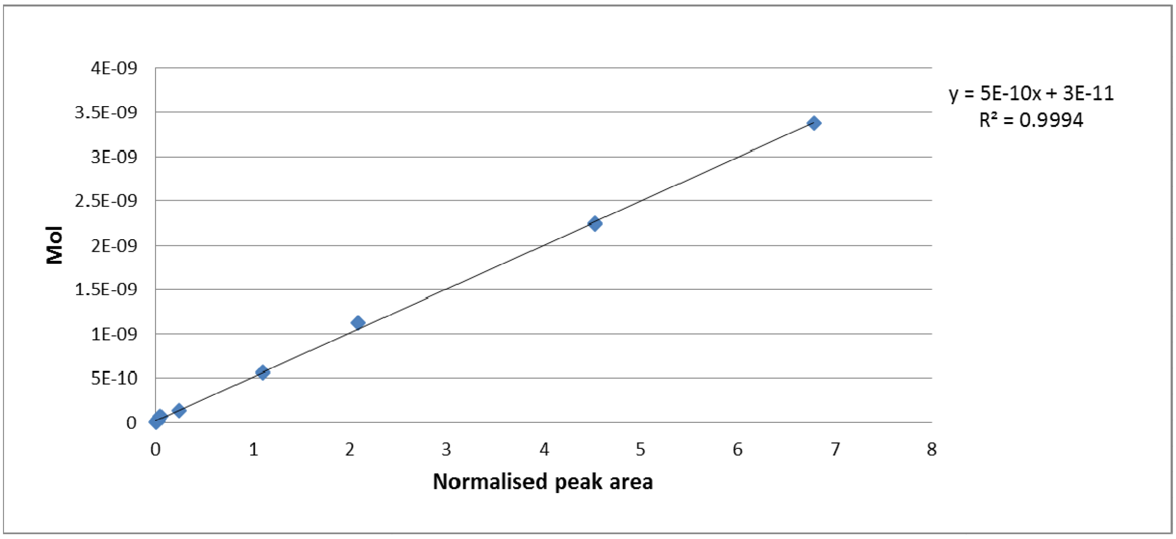

b)

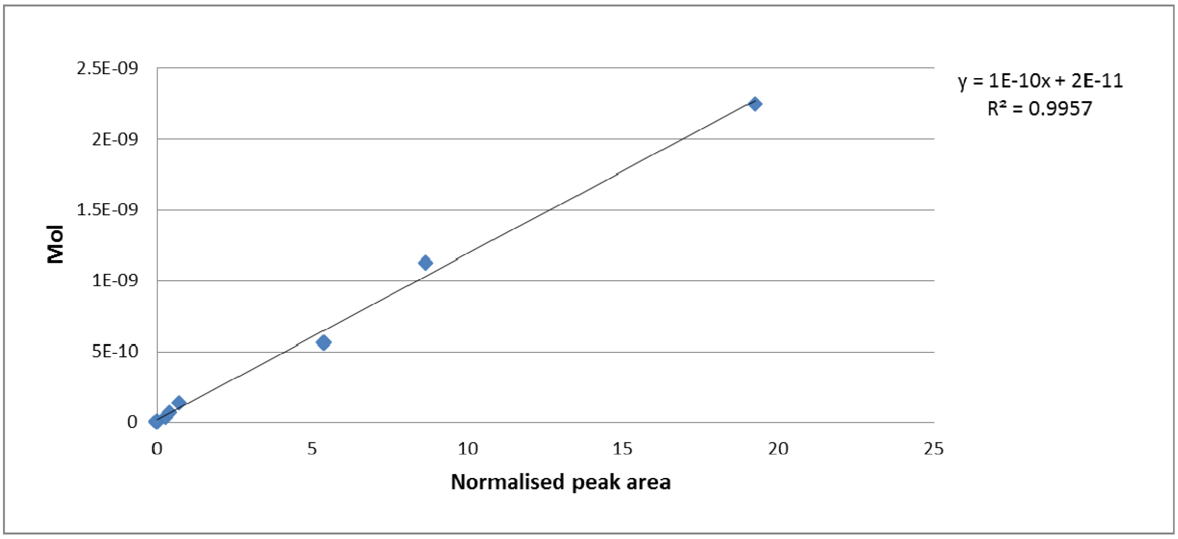

Figure S3 - Normalised calibration curves used to determine predicted intracellular levels of 1 and 5 by linear regression. Y axis: Moles of compound in the sample. X axis: Chromatogram peak area normalised by the peak area of the caffeine internal standard. a) Calibration curve for IOX1 **1;** b) Calibration curve for the *n*-octyl ester **5**.

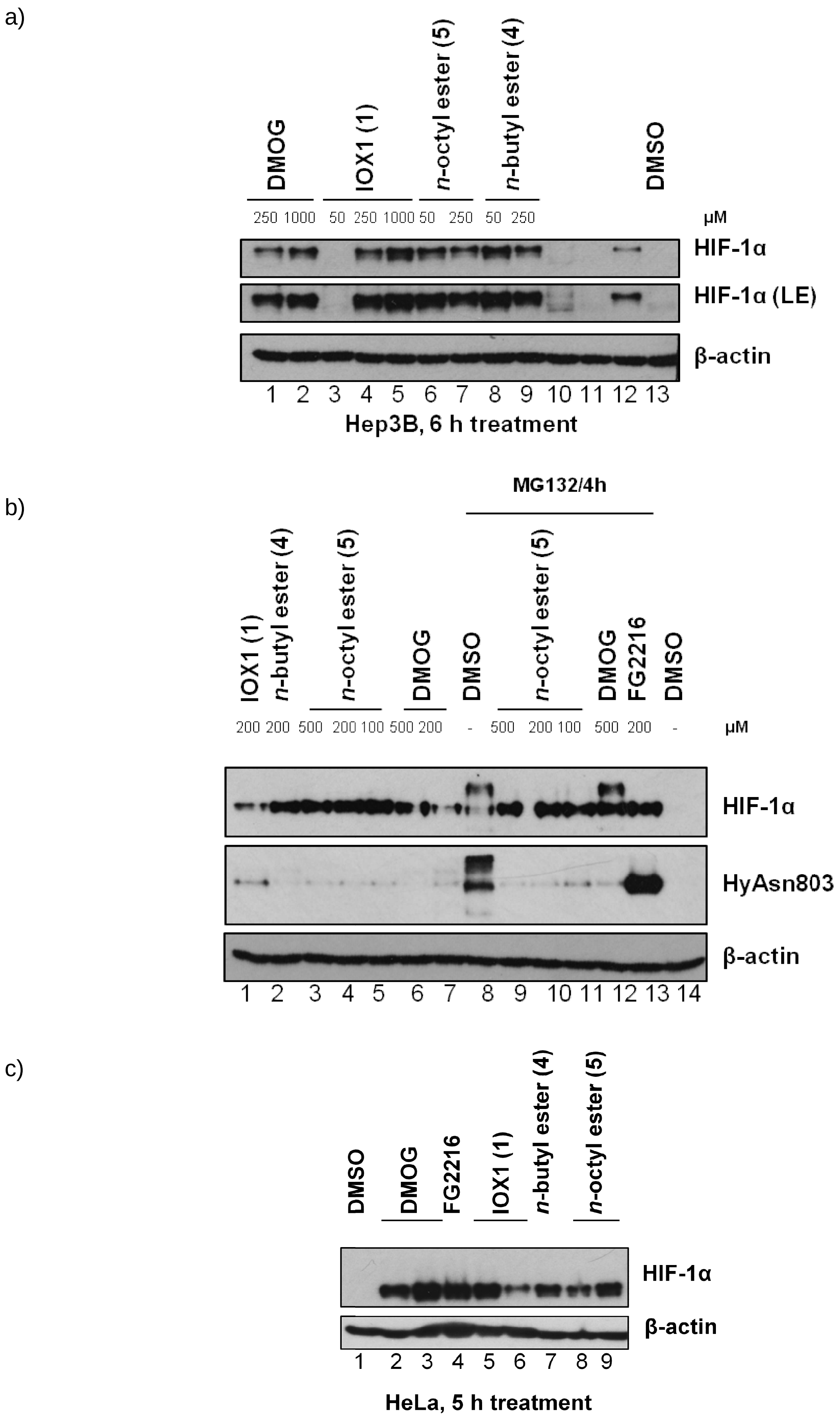
a)
DMOG
IOX1 (1)
n-octyl ester (5)
n-butyl ester (4)
DMSO
250 1000 50 250 1000 50 250 50 250
µM
HIF-1α
HIF-1α (LE)
β-actin
1 2 3 4 5 6 7 8 9 10 11 12 13
Hep3B, 6 h treatment
b)
MG132/4h
IOX1 (1)
n-butyl ester (4)
n-octyl ester (5)
DMOG
DMSO
n-octyl ester (5)
DMOG
FG2216
DMSO
200 200 500 200 100 500 200 - 500 200 100 500 200 -
µM
HIF-1α
HyAsn803
β-actin
1 2 3 4 5 6 7 8 9 10 11 12 13 14
c)
DMSO
DMOG
FG2216
IOX1 (1)
n-butyl ester (4)
n-octyl ester (5)
HIF-1α
β-actin
1 2 3 4 5 6 7 8 9
HeLa, 5 h treatment

d)

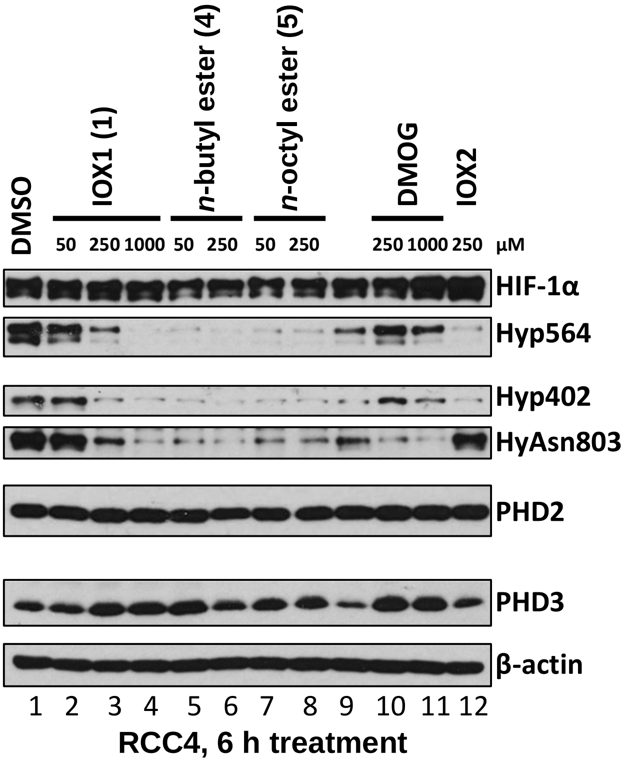

Figure S4 - Effects of IOX1 and IOX1 ester derivatives on HIF in human cell lines. Treatment of human cell lines Hep3B (a) and HeLa (b and c) with IOX1 and IOX1 ester derivatives (**4** and **5**) leads to upregulation of HIF-1α indicating PHD (HIF prolyl-hydroxylase) inhibition. Inhibition of HIF-1α hydroxylation at prolyl (Hyp) and asparaginyl (HyAsn) residues are observed in HIF-stabilised RCC4 cells (d). Note that **4** and **5** are both more active than IOX1 **1**, consistent with improved cell-penetration. A generic 2OG oxygenase inhibitor (DMOG/dimethyloxalylglycine), and PHD inhibitors (FG2216 and IOX2) were used as positive controls.[4] Note that the inhibition of HIF-1α asparaginyl hydroxylation was also observed implying the inhibition of factor inhibiting HIF (FIH) activity in cells. LE – long exposure.

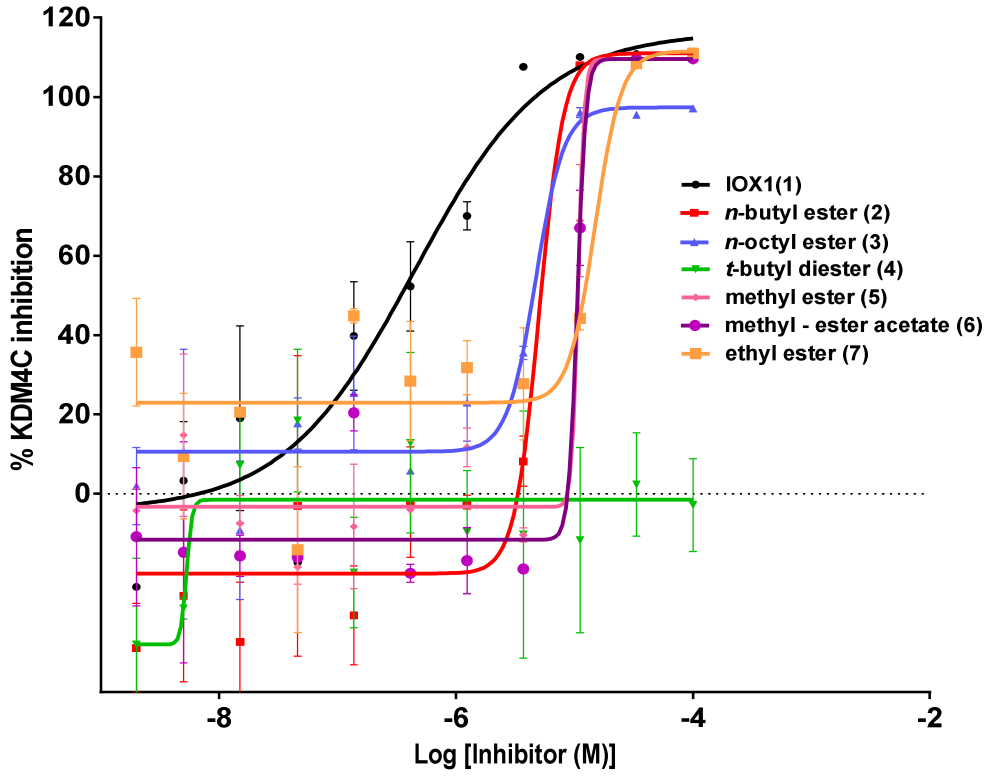

Figure S5 - KDM4C peptide turnover assayed by AlphaScreen. Evaluation of the in vitro Inhibition of KDM4C catalytic activity by IOX1 **1** and its ester derivatives. A counter screening with vehicle (DMSO) was used for normalisation. IC_{50} values are listed in Table 1. Values are mean ± SD, n=4. The procedure for the AlphaScreen assay is described in the biological procedures section.

a)

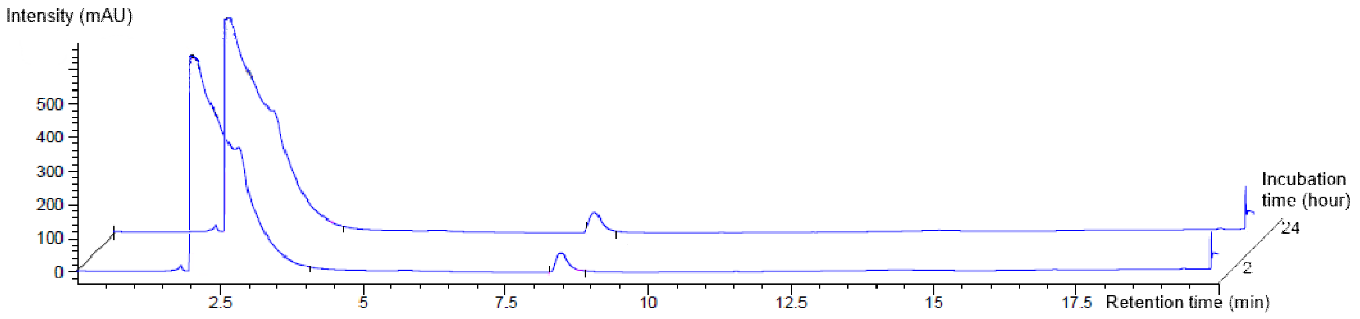

b)

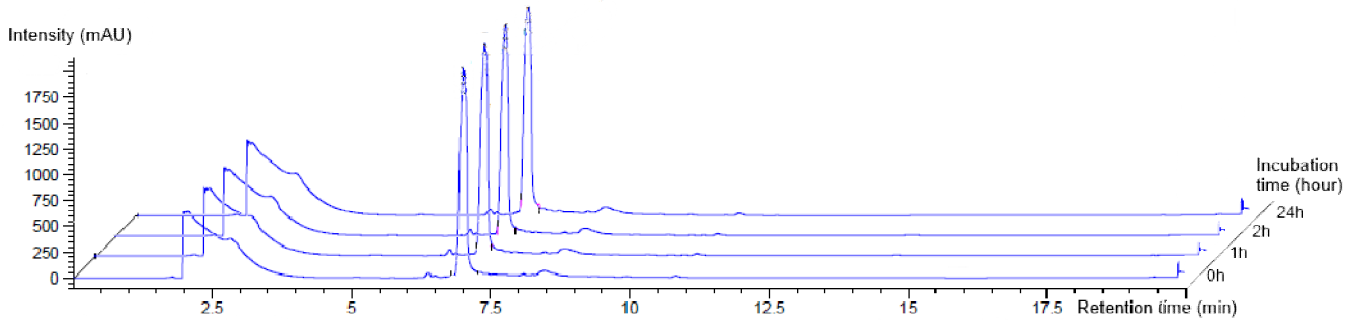

c)

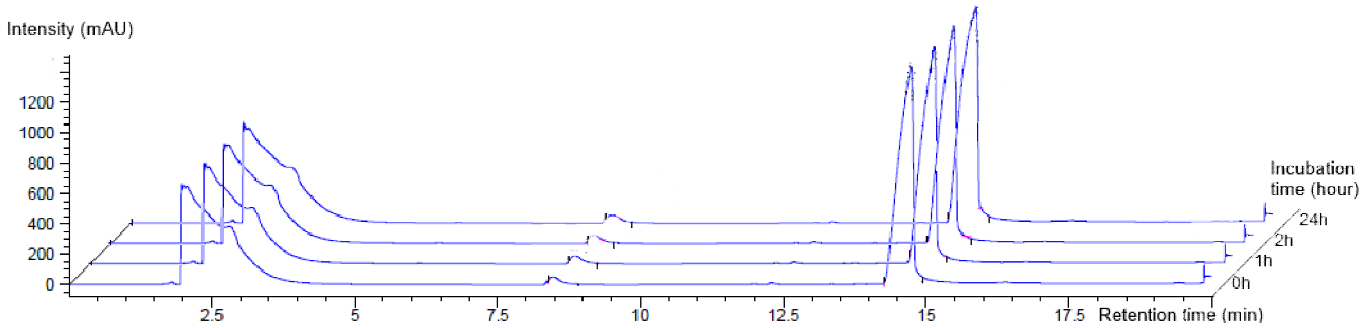

Figure S6 - Measurements of the stability of IOX1 1 and *n*-octyl ester 5 in the AlphaScreen buffer by LCMS. mAU: milliabsorbance units. X axis: time in minutes. Average retention times for observed species are: a) AlphaScreen buffer (50 mM HEPES pH 7.5 supplemented with 0.1% BSA, 0.01% Tween20, Fe(II) (10 µM), ascorbate (100µM) and 2OG (10 µM)), R_t = 2.0 and 8.5 minutes; b) IOX1 **1** in AlphaScreen buffer, R_t =7.0 minutes; c) Compound **5** in AlphaScreen buffer, R_t = 14.8 minutes. The procedure for the stability study in AlphaScreen buffer is described in the biological procedures section.

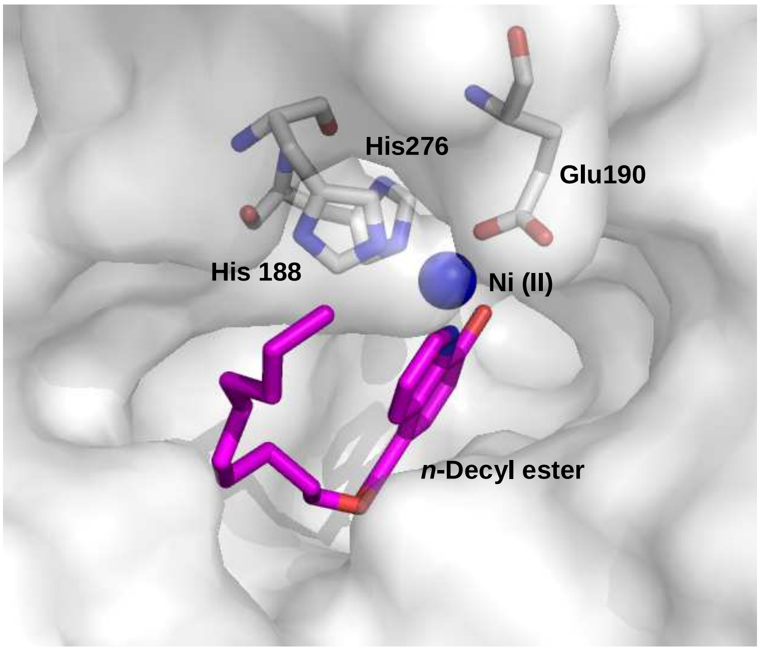

Figure S7 - Surface view of modelled *n*-decyl IOX1 ester derivative in the KDM4A active site. The docking simulation was based on a crystal structure of KDM4A bound to IOX1 (PDB code 3NJY).[3]

a)

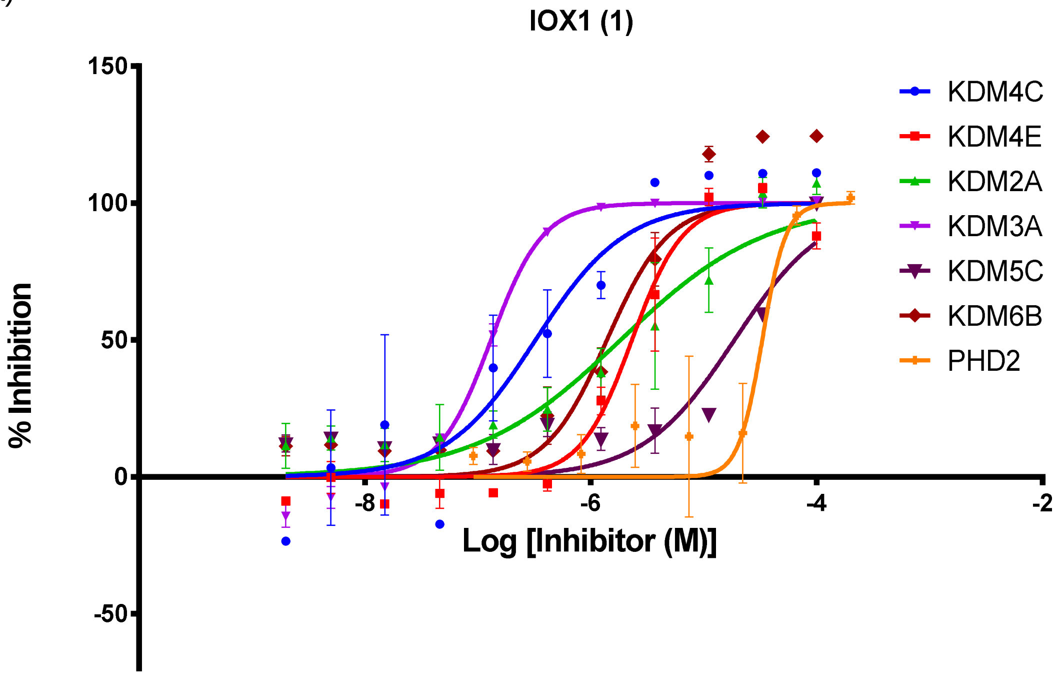
IOX1 (1)
150
100
50
0
-50
% Inhibition
-8
-6
-4
-2
Log [Inhibitor (M)]
KDM4C
KDM4E
KDM2A
KDM3A
KDM5C
KDM6B
PHD2

b)

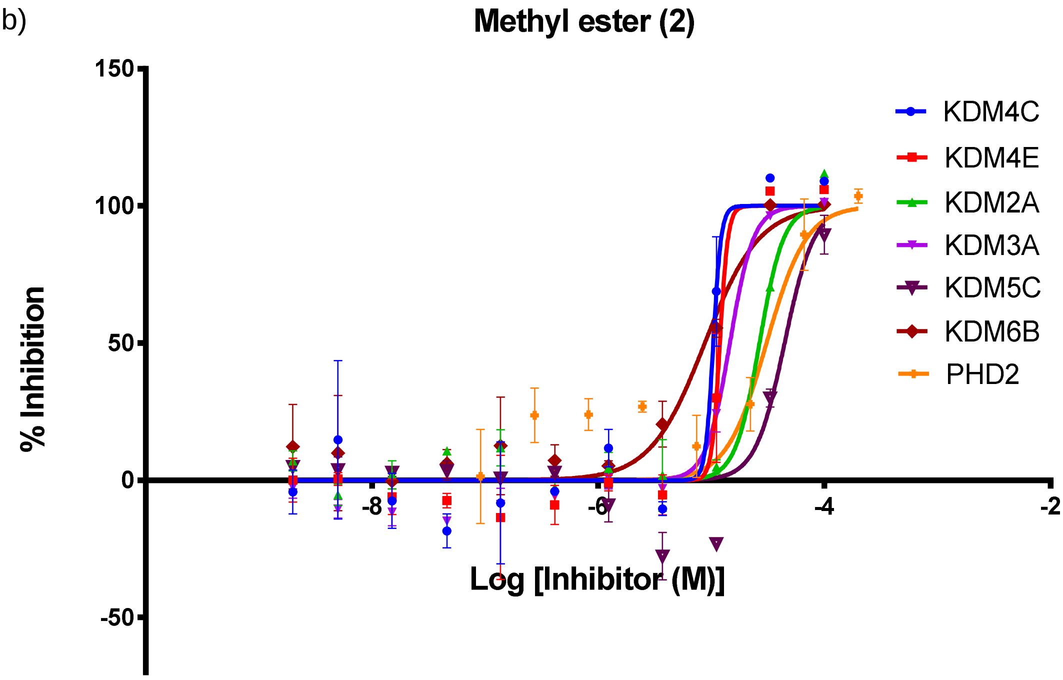
Methyl ester (2)
150
100
50
0
-50
% Inhibition
-8
-6
-4
-2
Log [Inhibitor (M)]
KDM4C
KDM4E
KDM2A
KDM3A
KDM5C
KDM6B
PHD2

c)

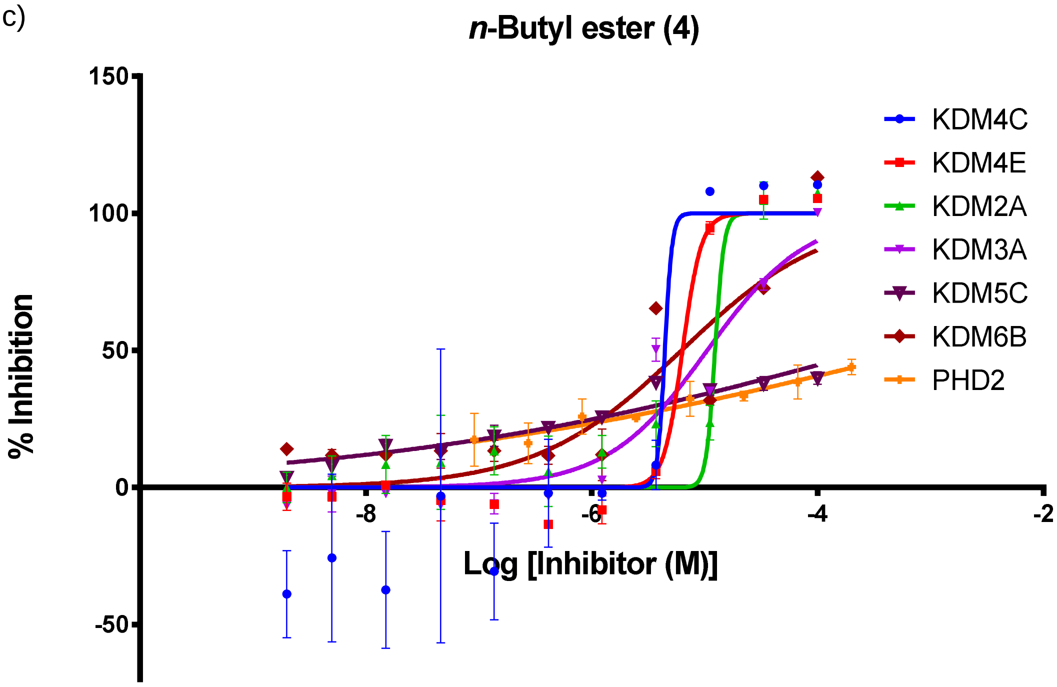

d)

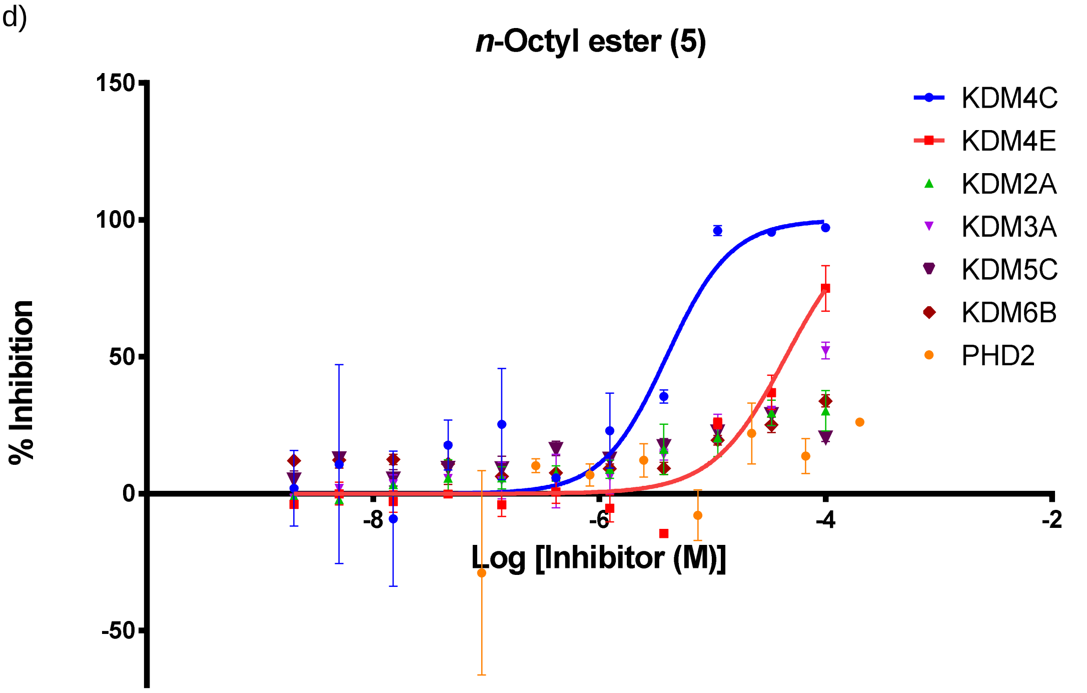

Figure S8 - In vitro selectivity for JmjC subfamilies. Evaluation of the in vitro inhibition of KDM4C, KDM4E, KDM2A, KDM3A, KDM5C, KDM6B and PHD2 assayed by AlphaScreen. Controls with DMSO were used for normalisation. IC_{50} values are listed in Table 3. Values are mean ± SD, n=4. The procedure for the AlphaScreen assay is described in the biological procedures section. a) Inhibition by IOX1 **1**; b) Inhibition by methyl ester **2;** c) Inhibition by *n*-butyl ester **4**; d) Inhibition by *n*-octyl ester **5.**

^{1}H NMR and HRMS for compounds 1 through 7

^{1}H NMR of Compound 1

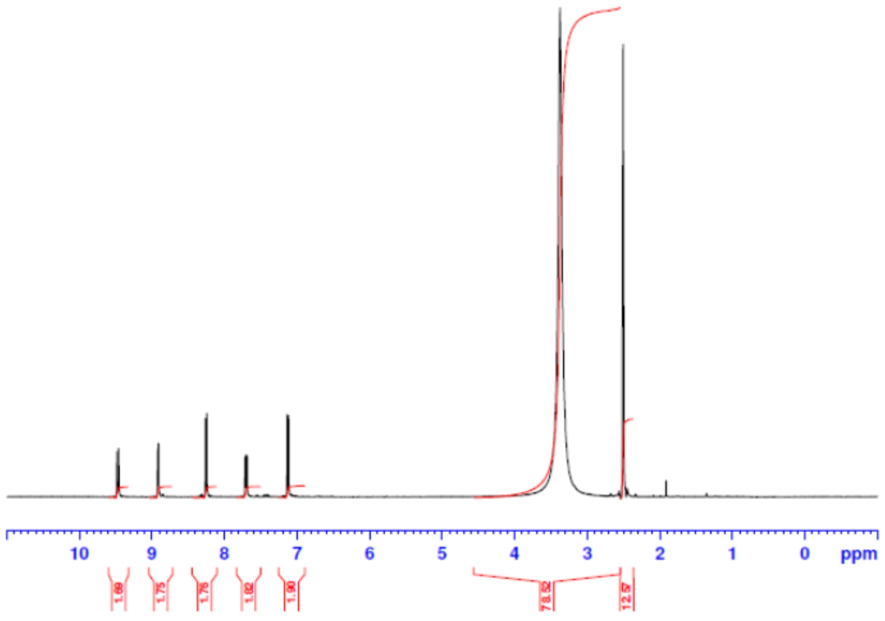

HRMS of Compound 1

Acquisition Parameter

Source Type	ESI	Ion Polarity	Negative	Set Nebulizer	2.0 Bar		
Focus	Not active			Set Dry Heater	180 °C		
Scan Begin	50 m/z	Set Capillary	4500 V	Set Dry Gas	10.0 l/min		
Scan End	1200 m/z	Set End Plate Offset	-500 V	Set Divert Valve	Source		

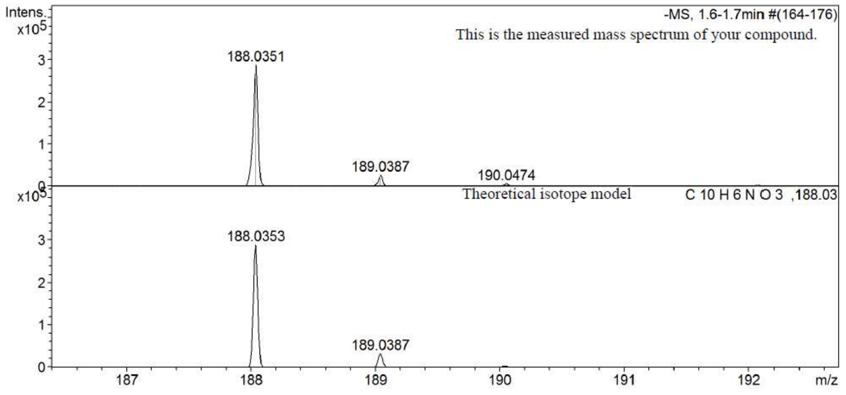

Meas. m/z	#	Formula	m/z	err [ppm]	Mean err [ppm]	rdb	N-Rule	e⁻ Conf	mSigma
188.0351									
	1	C 10 H 6 N O 3	188.0353	1.3	0.4	8.5	ok	even	11.50

^{1}H NMR of Compound 2

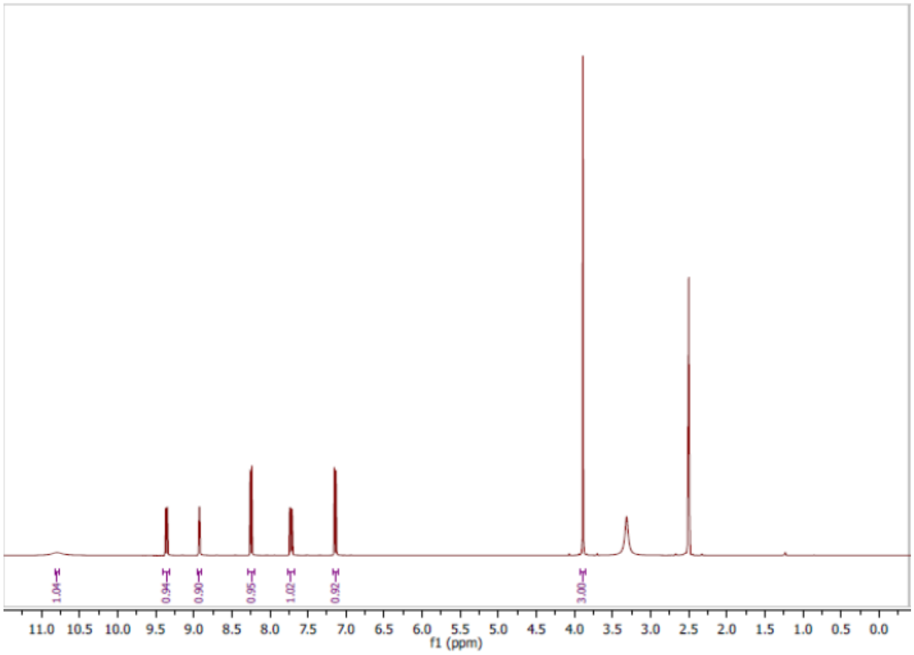

HRMS of Compound 2

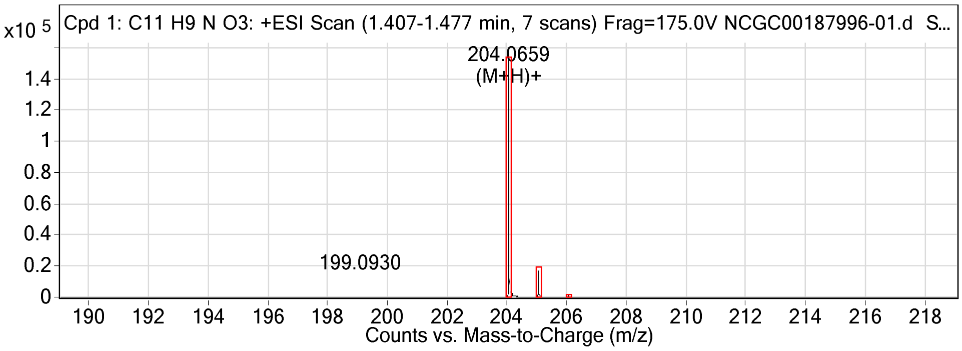

1H NMR of Compound 3

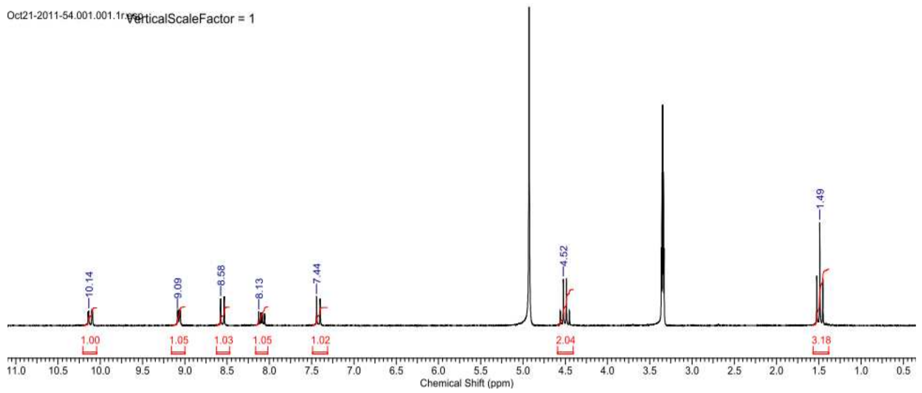

HRMS of Compound 3

Acquisition Parameter

Source Type	ESI	Ion Polarity	Positive			Set Nebulizer	2.0 Bar
Focus	Not active					Set Dry Heater	180 °C
Scan Begin	50 m/z	Set Capillary	4500 V			Set Dry Gas	10.0 l/min
Scan End	500 m/z	Set End Plate Offset	-500 V			Set Divert Valve	Source

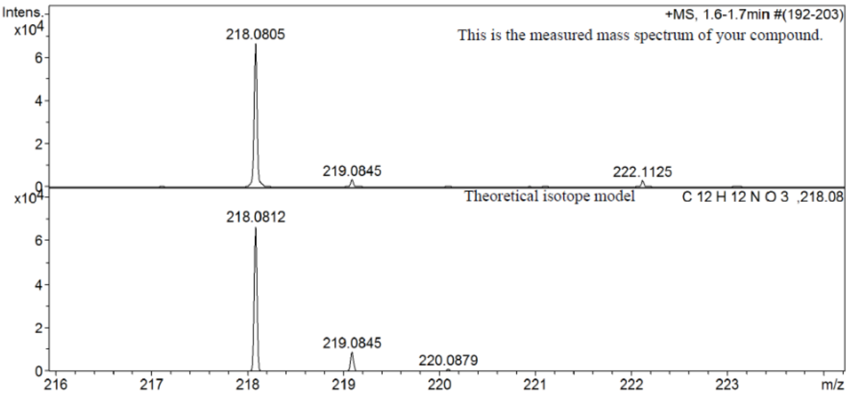

Meas. m/z	#	Formula	m/z	err [ppm]	Mean err [ppm]	rdb	e⁻ Conf	mSigma
218.0805								
	1	C 12 H 12 N O 3	218.0812	2.9	2.7	7.5	even	48.02

^{1}H NMR of Compound 4

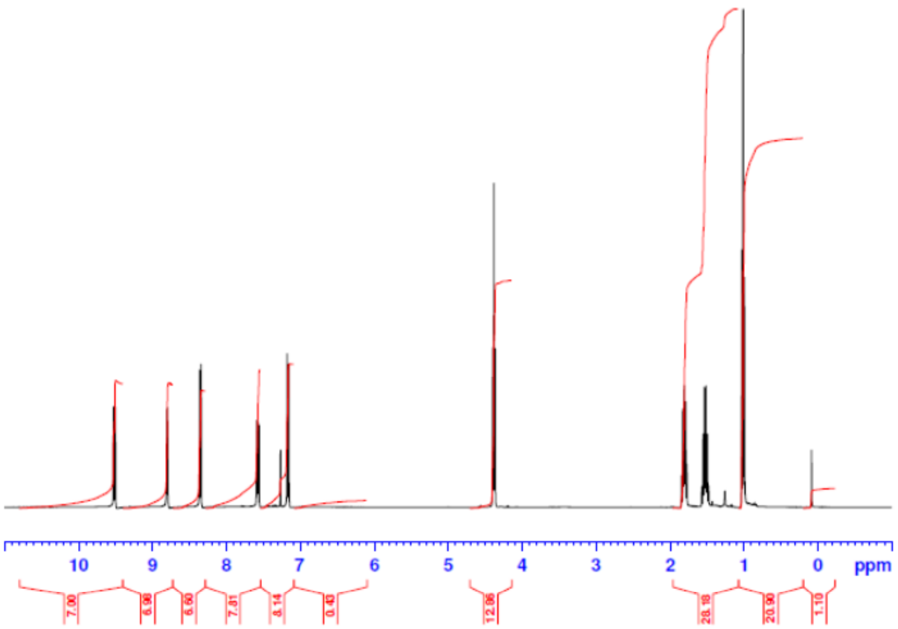

HRMS of Compound 4

Acquisition Parameter

Source Type	ESI	Ion Polarity	Positive	Set Nebulizer	2.0 Bar
Focus	Not active			Set Dry Heater	181 °C
Scan Begin	100 m/z	Set Capillary	4500 V	Set Dry Gas	10.0 l/min
Scan End	1000 m/z	Set End Plate Offset	-500 V	Set Divert Valve	Source

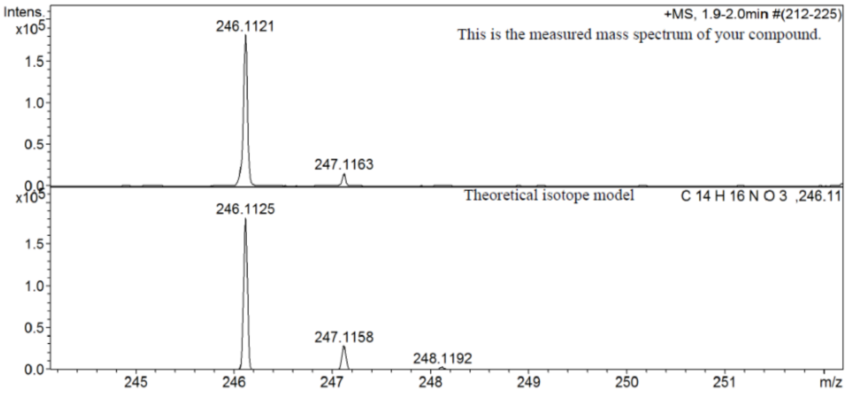

Meas. m/z	#	Formula	m/z	err [ppm]	Mean err [ppm]	rdb	e⁻ Conf	mSigma
246.1121								
	1	C 14 H 16 N O 3	246.1125	1.6	1.3	7.5	even	40.67

^{1}H NMR of Compound 5

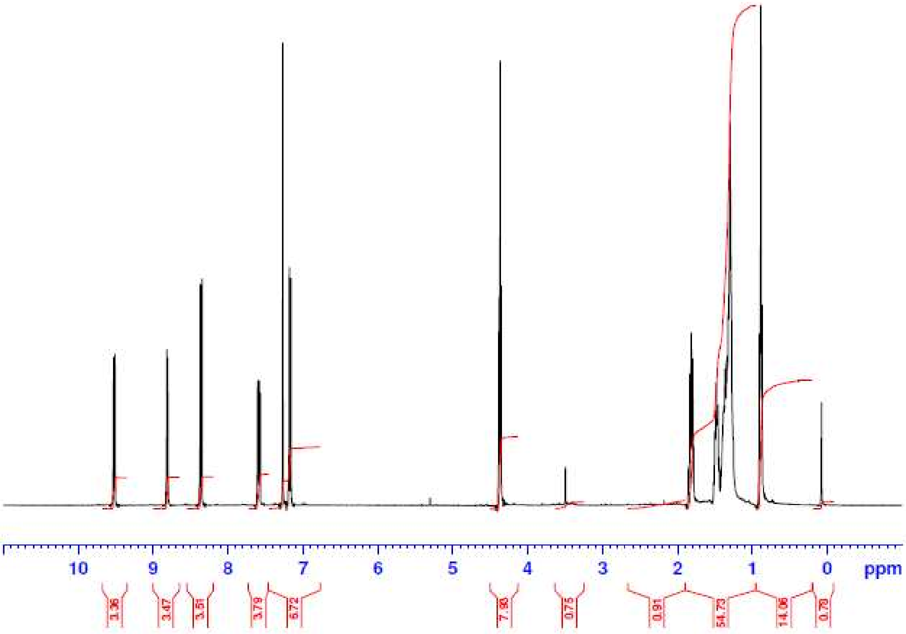

HRMS of Compound 5

Acquisition Parameter

Source Type	ESI	Ion Polarity	Positive	Set Nebulizer	2.0 Bar
Focus	Not active			Set Dry Heater	181 °C
Scan Begin	100 m/z	Set Capillary	4500 V	Set Dry Gas	10.0 l/min
Scan End	1000 m/z	Set End Plate Offset	-500 V	Set Divert Valve	Source

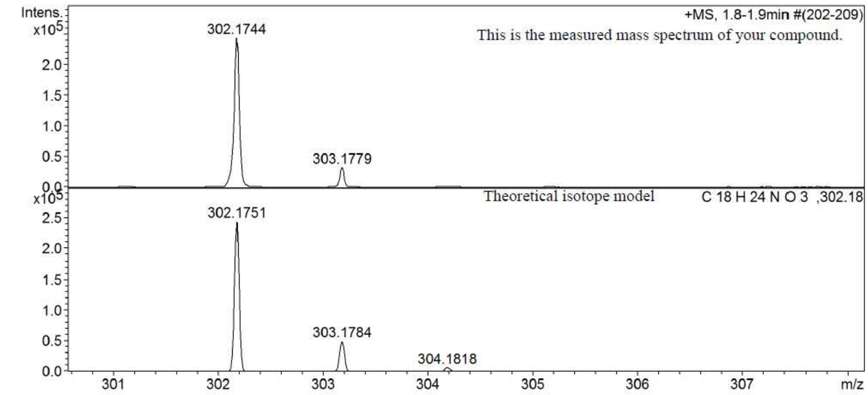

Meas. m/z	#	Formula	m/z	err [ppm]	Mean err [ppm]	rdb	e⁻ Conf	mSigma
302.1744								
	1	C 18 H 24 N O 3	302.1751	2.3	2.3	7.5	even	41.71

^{1}H NMR of Compound 6

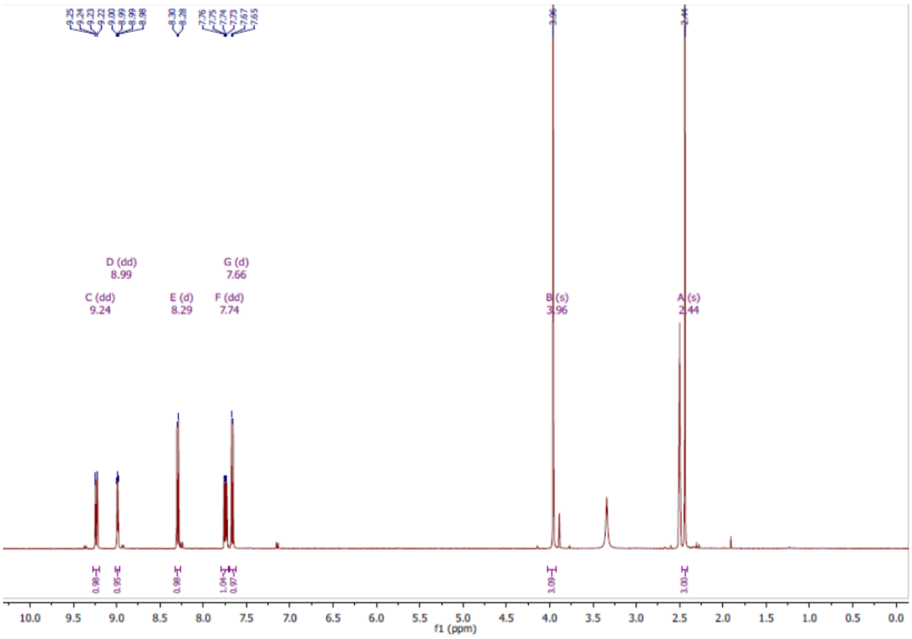

HRMS of Compound 6

Acquisition Parameter

Source Type	ESI	Ion Polarity	Positive	Set Nebulizer	2.0 Bar
Focus	Not active			Set Dry Heater	180 °C
Scan Begin	100 m/z	Set Capillary	4500 V	Set Dry Gas	10.0 l/min
Scan End	1000 m/z	Set End Plate Offset	-500 V	Set Divert Valve	Source

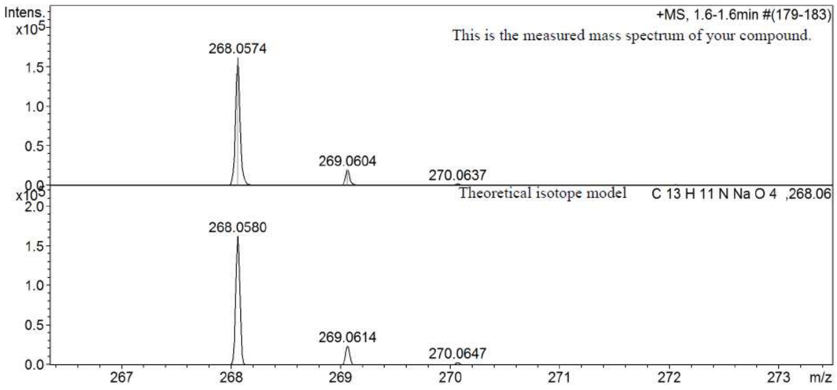

Meas. m/z	#	Formula	m/z	err [ppm]	Mean err [ppm]	rdb	e¯ Conf	mSigma
268.0574								
	1	C 13 H 11 N Na O 4	268.0580	2.4	2.5	8.5	even	12.92

^{1}H NMR of Compound 7

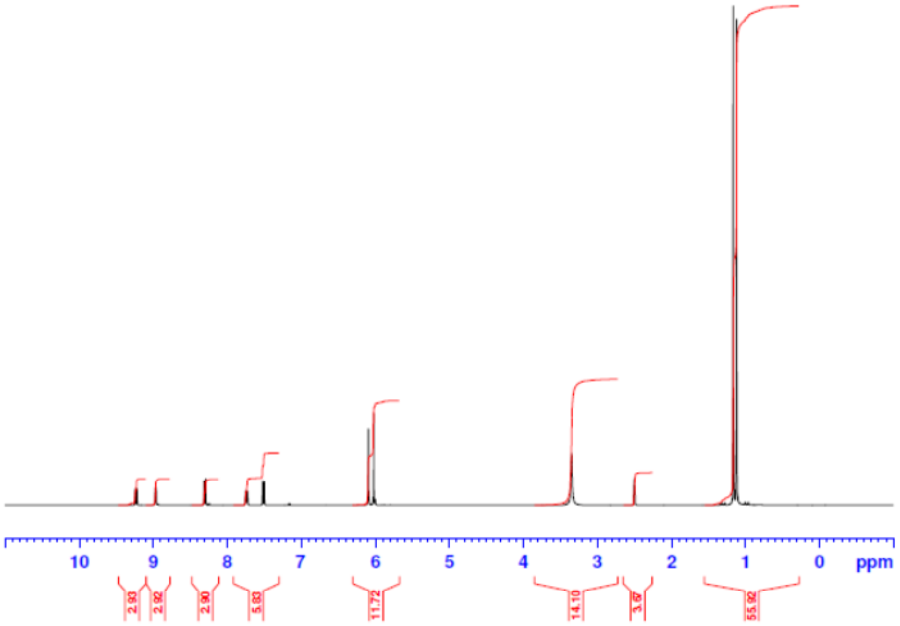

HRMS of Compound 7

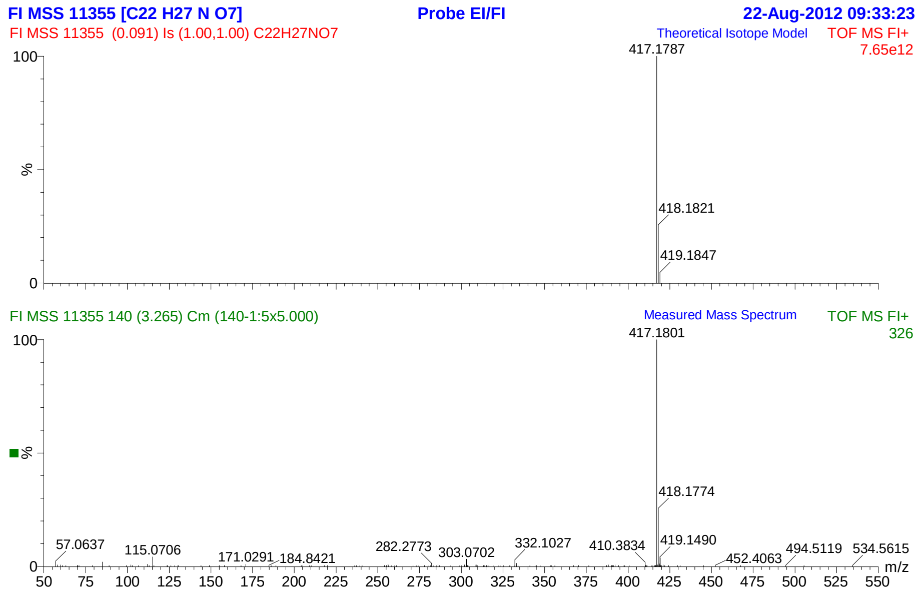

Figure S9 - ^{1}H NMR and HRMS for compounds 1 through 7.

Table S1 - The Gibbs free energy for binding of IOX1 and its ester derivatives to KDM4A as calculated by docking simulations

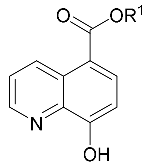

R^1	ΔG [Kcal/mol]
H	-7.05
CH_3	-6.37
CH_2CH_3	-6.06
$(CH_2)_2CH_3$	-6.69
$(CH_2)_3CH_3$	-6.85
$(CH_2)_5CH_3$	-6.63
$(CH_2)_7CH_3$	-6.75
$(CH_2)_9CH_3$	-6.64

References

[1] D. Rotili, M. Altun, A. Kawamura, A. Wolf, R. Fischer, I. K. H. Leung, M. M. Mackeen, Y.-M. Tian, P. J. Ratcliffe, A. Mai, B. M. Kessler, C. J. Schofield, *Chem. Biol.* **2011**, *18*, 642–54.

[2] O. N. F. King, X. S. Li, M. Sakurai, A. Kawamura, N. R. Rose, S. S. Ng, A. M. Quinn, G. Rai, B. T. Mott, P. Beswick, R. J. Klose, U. Oppermann, A. Jadhav, T. D. Heightman, D. J. Maloney, C. J. Schofield, A. Simeonov, *PLoS One* **2010**, *5*, e15535.

[3] A. Kawamura, A. Tumber, N. R. Rose, O. N. F. King, M. Daniel, U. Oppermann, T. D. Heightman, C. Schofield, *Anal. Biochem.* **2010**, *404*, 86–93.

[4] R. Chowdhury, J. I. Candela-Lena, M. C. Chan, D. J. Greenald, K. K. Yeoh, Y.-M. Tian, M. A. McDonough, A. Tumber, N. R. Rose, A. Conejo-Garcia, M. Demetriades, S. Mathavan, A. Kawamura, M. K. Lee, F. van Eeden, C. W. Pugh, P. J. Ratcliffe, C. J. Schofield, *ACS Chem. Biol.* **2013**, *8*, 1488–1496.

[5] L. Kruidenier, C. Chung, Z. Cheng, J. Liddle, K. Che, G. Joberty, M. Bantscheff, C. Bountra, A. Bridges, H. Diallo, D. Eberhard, S. Hutchinson, E. Jones, R. Katso, M. Leveridge, P. K. Mander, J. Mosley, C. Ramirez-Molina, P. Rowland, et al., *Nature* **2012**, *488*, 404–8.

[6] Y.-M. Tian, K. K. Yeoh, M. K. Lee, T. Eriksson, B. M. Kessler, H. B. Kramer, M. J. Edelmann, C. Willam, C. W. Pugh, C. J. Schofield, P. J. Ratcliffe, *J. Biol. Chem.* **2011**, *286*, 13041–51.

[7] J. P. Perdew, K. Burke, M. Ernzerhof, *Phys. Rev. Lett.* **1996**, *77*, 3865–3868.

[8] R. J. Hopkinson, A. Tumber, C. Yapp, R. Chowdhury, W. Aik, K. H. Che, X. S. Li, J. B. L. Kristensen, O. N. F. King, M. C. Chan, K. K. Yeoh, H. Choi, L. J. Walport, C. C. Thinnes, J. T. Bush, C. Lejeune, A. M. Rydzik, N. R. Rose, E. A. Bagg, et al., *Chem. Sci.* **2013**, *4*, 3110.

[9] G. M. Morris, D. S. Goodsell, R. S. Halliday, R. Huey, W. E. Hart, R. K. Belew, A. J. Olson, *J. Comput. Chem.* **1998**, *19*, 1639–1662.

[10] H. Kang, H. Choi, H. Park, *J. Chem. Inf. Model.* **2007**, *47*, 509–14.

[11] H. Choi, H. Kang, H. Park, *J. Cheminform.* **2013**, *5*, 8.
